# CEP Biomarkers as Potential Tools for Monitoring Therapeutics

**DOI:** 10.1371/journal.pone.0076325

**Published:** 2013-10-01

**Authors:** Kutralanathan Renganathan, Jiayin Gu, Mary E. Rayborn, John S. Crabb, Robert G. Salomon, Robert J. Collier, Michael A. Kapin, Carmelo Romano, Joe G. Hollyfield, John W. Crabb

**Affiliations:** 1 Cole Eye Institute, Cleveland Clinic Foundation, Cleveland, Ohio, United States of America; 2 Lerner Research Institute, Cleveland Clinic Foundation, Cleveland, Ohio, United States of America; 3 Department of Chemistry, Case Western Reserve University, Cleveland, Ohio, United States of America; 4 Retina Research, Novartis Institutes for Biomedical Research, Fort Worth, Texas, United States of America; 5 Departments of Ophthalmology and Molecular Medicine, Cleveland Clinic Lerner College of Medicine of Case Western Reserve University, Cleveland, Ohio, United States of America; University of Missouri-Columbia, United States of America

## Abstract

**Background:**

Carboxyethylpyrrole (CEP) adducts are oxidative modifications derived from docosahexaenoate-containing lipids that are elevated in ocular tissues and plasma in age-related macular degeneration (AMD) and in rodents exposed to intense light. The goal of this study was to determine whether light-induced CEP adducts and autoantibodies are modulated by pretreatment with AL-8309A under conditions that prevent photo-oxidative damage of rat retina. AL-8309A is a serotonin 5-HT_1A_ receptor agonist.

**Methods:**

Albino rats were dark adapted prior to blue light exposure. Control rats were maintained in normal cyclic light. Rats were injected subcutaneously 3x with 10 mg/kg AL-8309A (2 days, 1 day and 0 hours) before light exposure for 6 h (3.1 mW/cm^2^, λ=450 nm). Animals were sacrificed immediately following light exposure and eyes, retinas and plasma were collected. CEP adducts and autoantibodies were quantified by Western analysis or ELISA.

**Results:**

ANOVA supported significant differences in mean amounts of CEP adducts and autoantibodies among the light + vehicle, light + drug and dark control groups from both retina and plasma. Light-induced CEP adducts in retina were reduced ~20% following pretreatment with AL-8309A (n = 62 rats, *p* = 0.006) and retinal CEP immunoreactivity was less intense by immunohistochemistry. Plasma levels of light-induced CEP adducts were reduced at least 30% (n = 15 rats, *p* = 0.004) by drug pretreatment. Following drug treatment, average CEP autoantibody titer in light exposed rats (n = 22) was unchanged from dark control levels, and ~20% (*p* = 0.046) lower than in vehicle-treated rats.

**Conclusions:**

Light-induced CEP adducts in rat retina and plasma were significantly decreased by pretreatment with AL-8309A. These results are consistent with and extend previous studies showing AL-8309A reduces light-induced retinal lesions in rats and support CEP biomarkers as possible tools for monitoring the efficacy of select therapeutics.

## Introduction

There is growing evidence that disease mechanisms in age-related macular degeneration (AMD) involve oxidative stress and inflammation [1–4]. Such evidence includes accumulation of complement proteins in drusen [5–7], complement-associated AMD susceptibility genes [8–14], and many elevated inflammatory and immune process proteins in the macular AMD, Bruch’s membrane/choroid complex [15]. In addition, antioxidant vitamins selectively slow AMD progression [16], smoking increases the risk of AMD [17], and a host of oxidative modifications have been detected at elevated levels in AMD ocular tissues and plasma [4]. Among oxidative modifications, carboxyethylpyrrole (CEP) adducts, derived from fragmentation of docosahexaenoate (DHA)-containing lipids, have been compellingly linked with AMD pathology. CEP adducts are elevated in drusen and in the AMD, Bruch’s membrane/choroid/retinal pigment epithelium (RPE) complex [5]. CEP adducts simulate angiogenesis *in vivo* [18,19], suggesting a role in neovascular AMD, and mice immunized with CEP adducts develop a dry AMD-like phenotype [20]. Analyses of 1400 AMD and control donors have demonstrated that CEP adducts and autoantibodies are elevated in AMD plasma and offer AMD biomarker potential [21,22]. Combined CEP proteomic and genomic biomarker measurements appear more effective in assessing AMD risk than either approach alone [22].

CEP oxidative modifications are elevated in other animal models exhibiting phenotypic similarities with AMD, including superoxide dismutase 2 ribozyme knockdown mice [23] and rodents exposed to intense green [24] and blue [25] light. Green light-induced CEP adducts in rat retina can be reduced by pretreatment with zinc oxide [24]. The purpose of this study was to determine whether the formation of blue light-induced CEP adducts and autoantibodies are modulated by AL-8309A under conditions employed in two recent studies that demonstrated AL-8309A prevents light-induced retinal lesions in rats, and decreases microglia activation and complement deposition in rat retina [25,26]. Here we show that light-induced CEP adducts in rat retina and plasma are decreased by pretreatment of rats with AL-8309A, a serotonin 1A (5-hydroxytryptamine or 5-HT) receptor agonist.

## Methods

### Ethics Statement

All animal procedures were performed at Alcon Research, Ltd, and adhered to the ARVO Statement for the Use of Animals in Ophthalmic and Vision Research. Animal procedures in this study were approved by and carried out under the supervision of the Alcon Animal Care and Use Committee (Permit Number 2007-419-Collier); all efforts were made to minimize animal suffering.

### Animal Procedures

Male Sprague-Dawley rats (weight range 300-450 g) were exposed to blue light with or without prior AL-8309A drug treatment as previously described [25,26]. AL-8309A was obtained from Dainippon Sumitomo, Osaka, Japan. Rats were dark adapted 24 hours prior to blue light exposure for 6 hours (3.1 mW/cm^2^, Philips fluorescent lamps [F40/BB], half-amplitude band pass = 435-465 nm). Control rats were housed under broad-band fluorescent (Sylvania Cool White, 45 ft-c) cyclic light (12 hours light, 12 hours dark) then dark adapted 24 hours prior to sacrifice. Rats were injected 3x subcutaneously with vehicle (sterile 0.9% sodium chloride) or with 10 mg/kg AL-8309A (2 days, 1 day and 0 hours) before light exposure. Animals were sacrificed at the same time each day immediately following light exposure; eyes were excised and blood collected by cardiac puncture under dim red illumination.

### Retina Preparations

For Western analyses, rat retinas were isolated without RPE or choroid [27] under dim red illumination within 2 min of death, rinsed in PBS containing 2 mM diethylenetriamine-pentaacetic acid and 100 µM butylated hydroxytoluene (BHT), frozen in tubes in liquid nitrogen, wrapped in aluminum foil and shipped on dry ice to the Cole Eye Institute. Under dim red illumination, retinas were thawed, lipids were extracted with chloroform/methanol, and the aqueous phase was vacuum dried for protein extraction. Protein was extracted by vortexing in 300 µl of 60 mM TrisCl buffer containing 2% SDS, 10 mM DTT, 100 µM BHT, and 2 mM EDTA, followed by centrifugation at 60,000 g for 7 min. The supernatant was removed and the extraction repeated 2x with 150 µl of the above extraction buffer. The supernatants were combined, flushed with argon, and stored at -80 °C until analysis for CEP adducts.

### Plasma Preparations

Rat blood was collected in tubes containing EDTA and plasma was prepared immediately [21,22]. Plasma was aliquoted containing the antioxidant BHT (22 µg/ml plasma), protease inhibitors (Sigma-Aldrich product P8340, 10 µl/ml plasma), then flushed with argon, quench frozen in liquid nitrogen and stored under argon at -80 °C until analysis for CEP adducts and autoantibodies [21,22]. All samples were frozen and thawed only once.

### Experimental Design and Analytical Procedures

CEP adducts and autoantibodies were quantified in three groups of animals: light exposed rats pretreated with AL-8309A; light exposed rats pretreated with vehicle (sterile 0.9% sodium chloride, Sigma-Aldrich); and dark controls (rats without light or vehicle treatments either with or without drug pretreatment). Animal procedures were performed at Alcon Research, Ltd and analytical procedures at the Cole Eye Institute. All analytical procedures were performed without prior knowledge of the animal group from which the specimens originated. The origin of all specimens was revealed only after the results of the blinded analytical procedures were announced.

CEP adducts and CEP autoantibodies were assayed using previously described Western blot, ELISA and immunohistochemistry methods [5,20–22]. Briefly for Western blots [5,21,22], protein was solubilized in Laemmli SDS sample buffer [28]. To obtain equal sample loading per gel lane (~15 µg), protein concentrations were measured by the Bradford assay [29], a preliminary SDS-PAGE analysis was performed, the gel was stained with Coomassie blue and scanned with a GS-710 imaging densitometer (BioRad), and Coomassie blue staining intensity was quantified with Quantity One software (BioRad). Sample amounts were then adjusted to obtain equal staining intensity in each gel lane, and verified by subsequent Coomassie stained SDS-PAGE prior to Western analysis. This global method for equalizing sample amounts has been used in several previous studies [5,22,30,31]. Others have demonstrated linearity of transfer from gel to membrane over the range of sample amounts used in this study, further supporting Coomassie blue staining as a loading control for Western analysis [32]. Western blot CEP immunoreactivity was detected with anti-CEP monoclonal antibody and quantified by densitometry and Quantity One software (BioRad). Western blots utilized CEP modified human serum albumin (CEP-HSA) as a positive control. CEP autoantibody titer was determined in rat plasma using a previously described ELISA procedure for quantifying CEP autoantibodies in human plasma [21].

For immunocytochemistry, rat eyes were enucleated immediately after light exposure, anterior segments removed and posterior eye cups were fixed in 4% paraformaldehyde in PBS for 4 h at 4 °C [33]. The eye cups were then cryoprotected in 30% sucrose overnight, embedded in optimal cutting temperature compound, and cryosections (16 µm) were prepared and stored at -80 °C until analysis [33]. Sections were probed with rabbit polyclonal anti-CEP antibody [5,20,21]. A total of 15 eyes were analyzed from 15 rats, including 5 light exposed rats pretreated with AL-8309A, 5 light exposed rats pretreated with vehicle and 5 dark control animals.

### Statistics

Continuous measures were summarized using means and standard deviations calculated from log_10_ transformed optical density and antibody titer data normalized to the mean dark control per analysis. Outliers were eliminated from normalized datasets using Dixon’s test for n ≤ 30 measurements [34,35] and the interquartile range for n > 30 measurements [36]. The distribution of normalized datasets was evaluated using the Shapiro-Wilk Normality Test [37]. Normalized CEP biomarker concentrations among the three groups (dark control, light + vehicle, and light + drug) were compared by single factor ANOVA using Excel 2010 (Microsoft Office). CEP biomarker differences between each pair of groups were further evaluated by the 2-sided *t*-test (Excel 2010). When ANOVA results supported a significant difference among the 3 groups, a Bonferroni adjustment (0.05 divided by 3) was applied, setting p ≤ 0.017 as the criteria of statistical significance for the t-test. Mean log values were transformed to linear scale for reporting.

## Results

### Blue Light Elevates and AL-8309A Reduces CEP Adducts in Rat Retina

**Figure 1:**
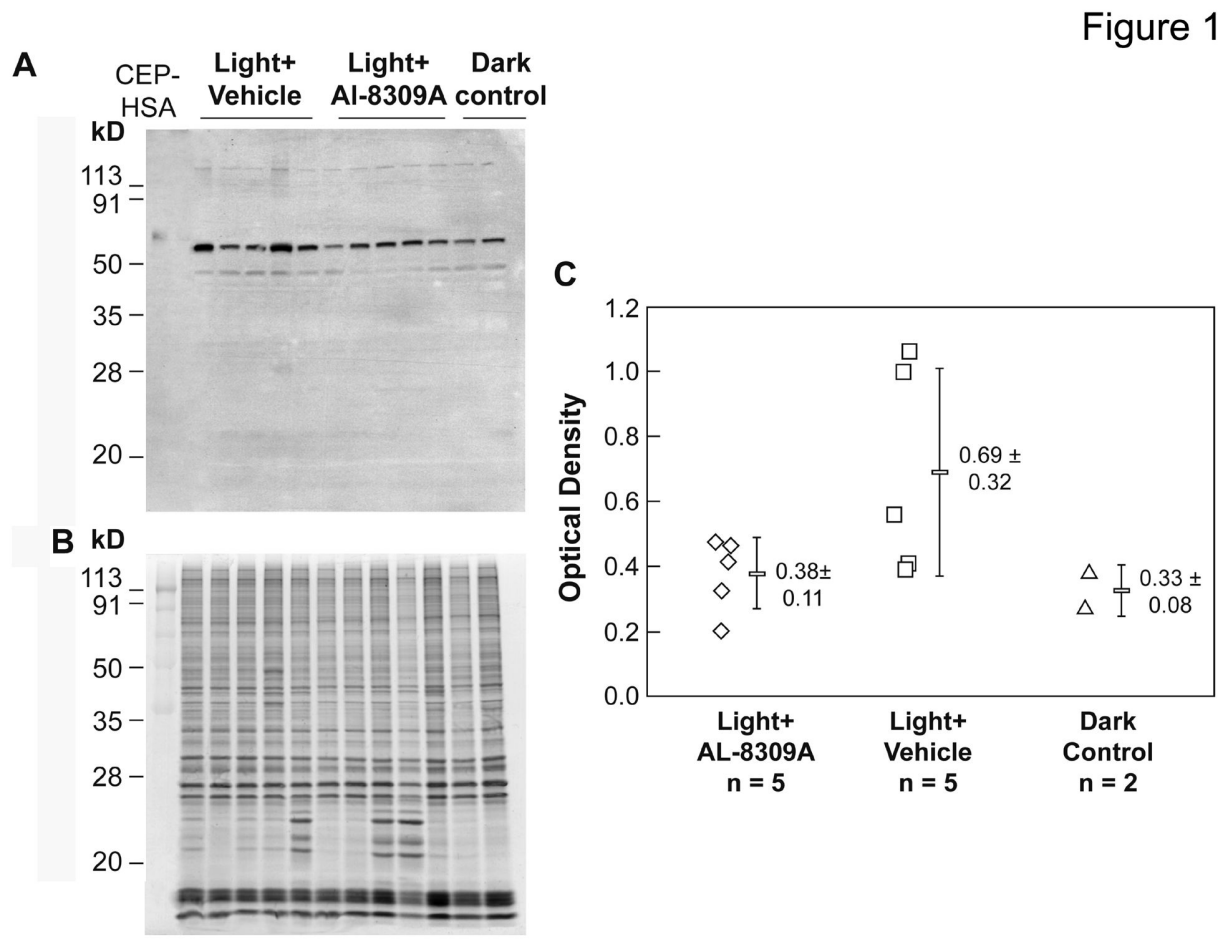
CEP immunoreactivity in rat retina. A summary is shown of CEP adduct levels in rat retina following blue light exposure with or without pretreatment with AL-8309A (from Western analyses in Figure S2). Log10 transformed CEP optical density measurements were normalized to the mean dark control per analysis, outliers were eliminated and average ratios were transformed to linear scale. Δ represents the difference in average ratios between animals groups, the p-values are from the 2-sided t-test and error bars reflect standard deviation. The number of animals assayed in each group is indicated (n).

**Figure 2:**
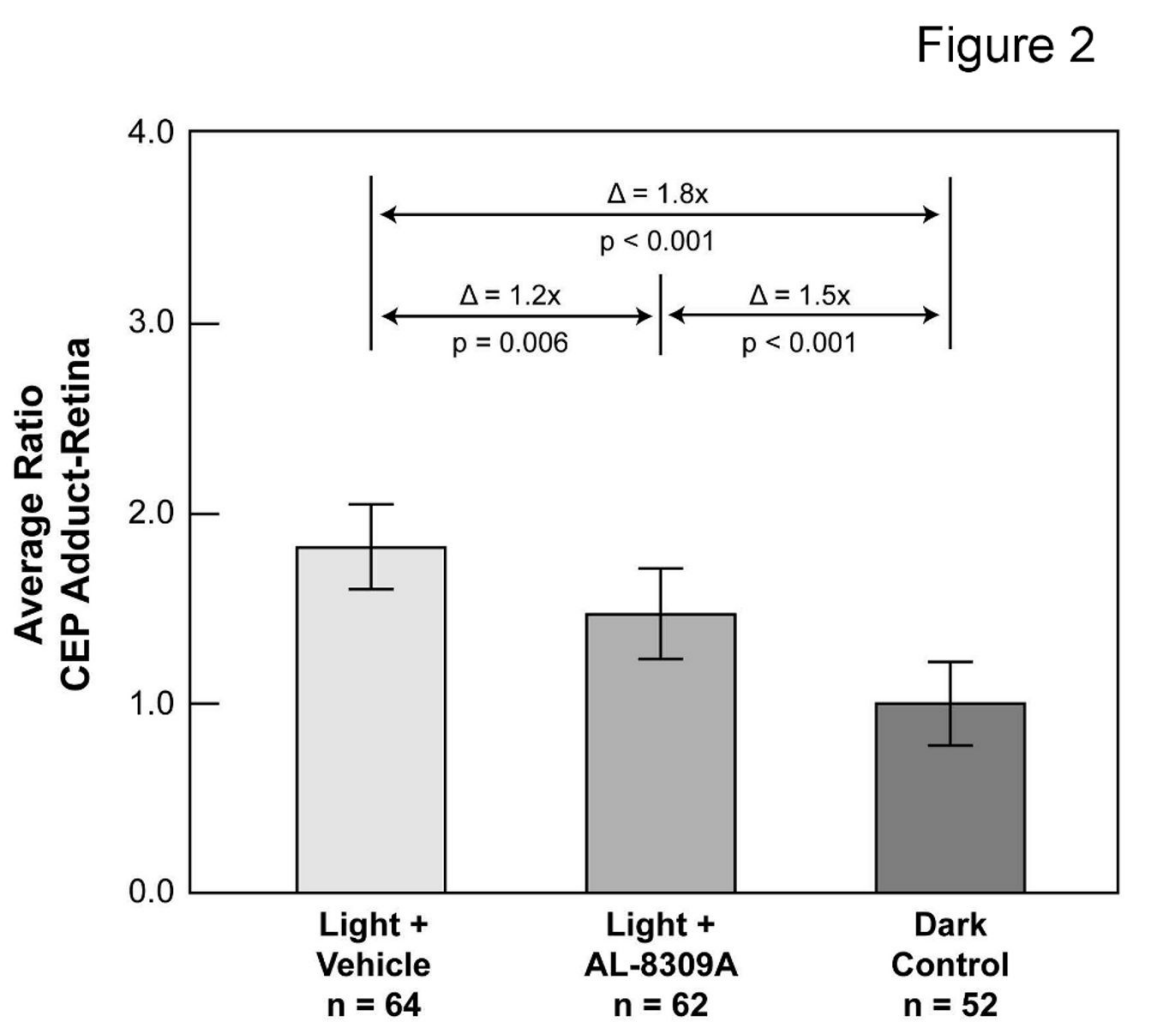
Pretreatment with AL-8309A reduces light-induced CEP immunoreactivity in rat retina. A summary is shown of CEP adduct levels in rat retina following blue light exposure with or without pretreatment with AL-8309A (from Western analyses in Figure S2). Log_10_ transformed CEP optical density measurements were normalized to the mean dark control per analysis, outliers were eliminated and average ratios were transformed to linear scale. Δ represents the difference in average ratios between animals groups, the p-values are from the 2-sided t-test and error bars reflect standard deviation. The number of animals assayed in each group is indicated (n).

Western analysis with anti-CEP monoclonal antibody was used to quantify CEP adducts in rat retinal homogenates following *in vivo* blue light exposure with or without pretreatment with AL-8309A as illustrated in Figure 1 and Figure S1. CEP Western analyses of rat retina from three separate experiments involving 53-64 animals are presented in Figure S2 (available on line). Figure 2 presents an overall summary of the normalized immunoblot results from the retina after removal of outlier measurements (~4.3% of 300 total measurements). Outlier elimination reduced dark control animals from 64 to 62 and light + drug animals from 53 to 52 but did not impact the number of animals in the light + vehicle group (n = 64). All three datasets exhibited a normal distribution by the Shapiro-Wilk Normality Test and their means were significantly different by ANOVA (F = 33.7, F critical = 3.03, *p* < 0.001). Data variability was 21.6% relative standard deviation (RSD) for the dark control group (including outliers), and 16.6% RSD for the light + drug group, and 12.3% RSD for the light + vehicle group (both excluding outliers). The results in Figure 2 shows that blue light exposure elevated CEP immunoreactivity in retina ~1.8-fold (t-test *p* < 0.001) in vehicle-treated animals relative to rats not exposed to intense light. In contrast, pretreatment with AL-8309A prior to light exposure resulted in a smaller increase of ~1.5-fold (t-test p < 0.001) relative to the dark control. Pretreatment with AL-8309A reduced CEP immunoreactivity in retinal lysates ~20% (t-test *p* = 0.006) relative to light exposed, vehicle-treated rats. Treatment with AL-8309A in the absence of light exposure had no apparent effect on the level of CEP adducts in rat retina (Table S1).

**Figure 3:**
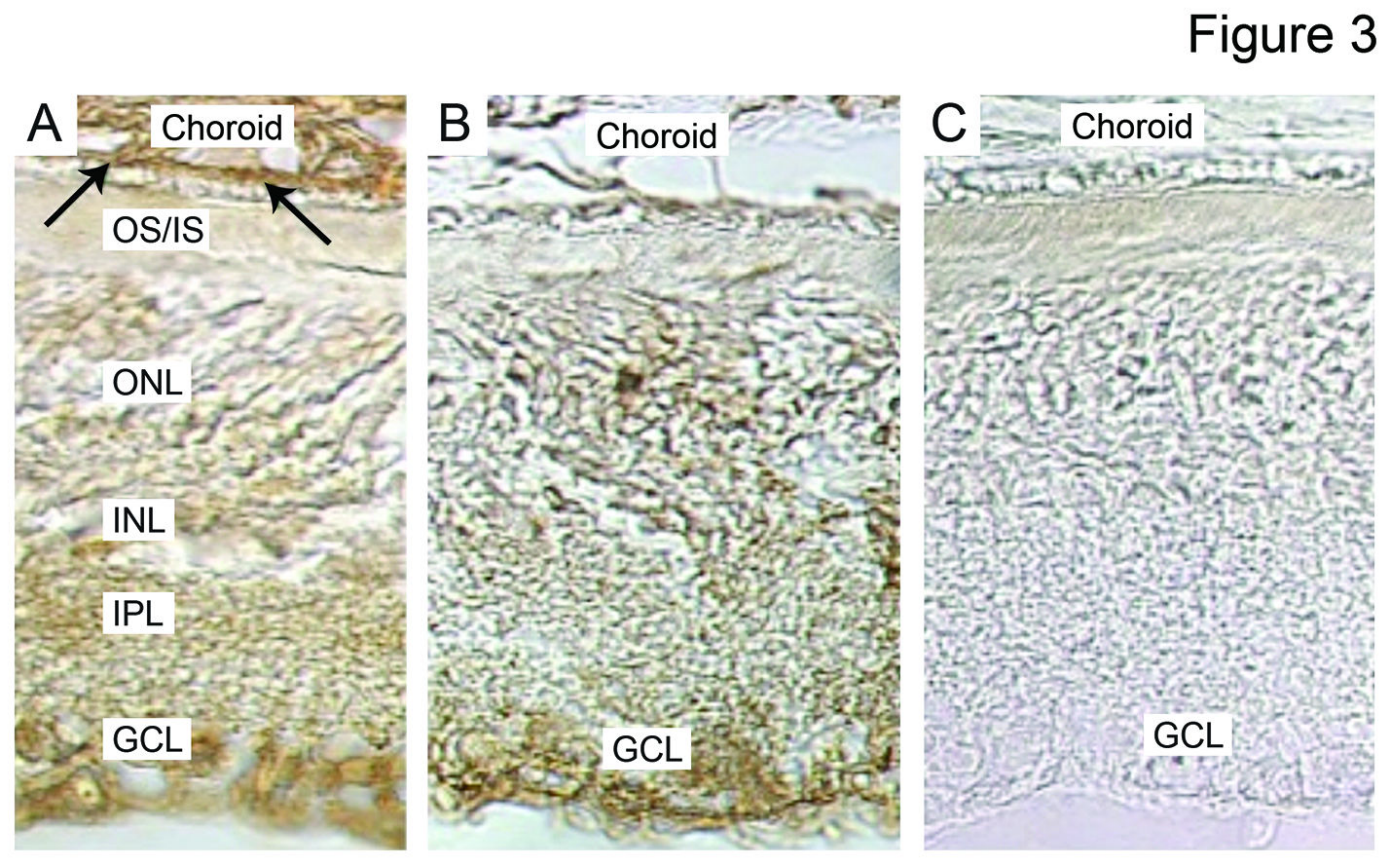
CEP Immunohistochemistry. Representative immunohistochemical analyses of posterior globe sections with rabbit anti-CEP polyclonal antibody are shown from light exposed rats pretreated with vehicle (A), or pretreated with AL-8309A (B), or dark control animals (C). Animals pretreated with vehicle and light show intense CEP-labeling in the ganglion cell layer (GCL), inner plexiform layer (IPL) of the retina, Bruch’s membrane (arrows) and choroid. In animals pretreated with AL-8309A, the GCL layer and Bruch’s membrane exhibit reduced CEP immunoreactivity and labeling is absent in the choroid. The dark control exhibits minimal to no CEP immunoreactivity. OS/IS, outer segments/inner segments; ONL, outer nuclear layer; INL, inner nuclear layer.

Immunohistochemical analyses of rat eyes following blue light exposure with and without pretreatment with AL-8309A were consistent with the Western results of retina. Compared with the dark control, which lacked significant CEP immunoreactivity, light exposed rats without AL-8309A treatment exhibited intense CEP labeling in the retina, choroid and Bruch’s membrane (Figure 3). Rats treated with AL-8309A prior to light exposure exhibited less intense CEP labeling in the retina and Bruch’s membrane and no apparent CEP labeling of the choroid (Figure 3).

### Blue Light Elevates and AL-8309A Reduces CEP Adducts in Rat Plasma

**Figure 4:**
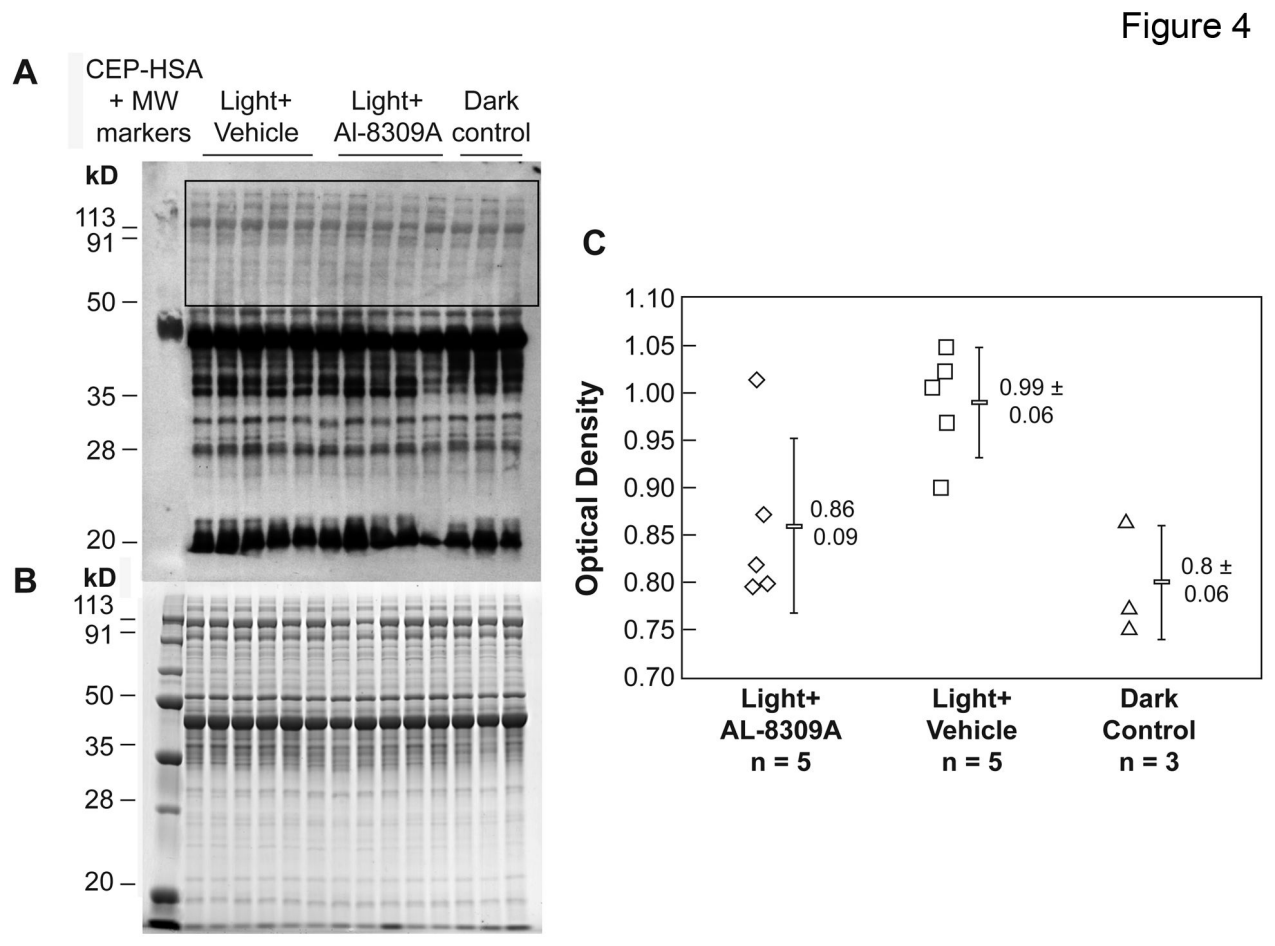
CEP immunoreactivity in rat plasma. (A) A representative CEP Western blot of rat plasma is shown following in vivo blue light exposure with or without pretreatment with AL-8309A. Each lane represents one animal. CEP modified human serum albumin (CEP-HSA, 2 ng) was used as a positive control. (B) A representative Coomassie Blue stained gel before blotting is shown; Supporting Figure S3 shows that approximately equal amounts of protein were applied per lane for Western analysis. (C) CEP immunoreactivity was quantified by densitometry as shown in the graphs where error bars represent standard deviation. The number of animals assayed in each group is indicated (n).

**Figure 5:**
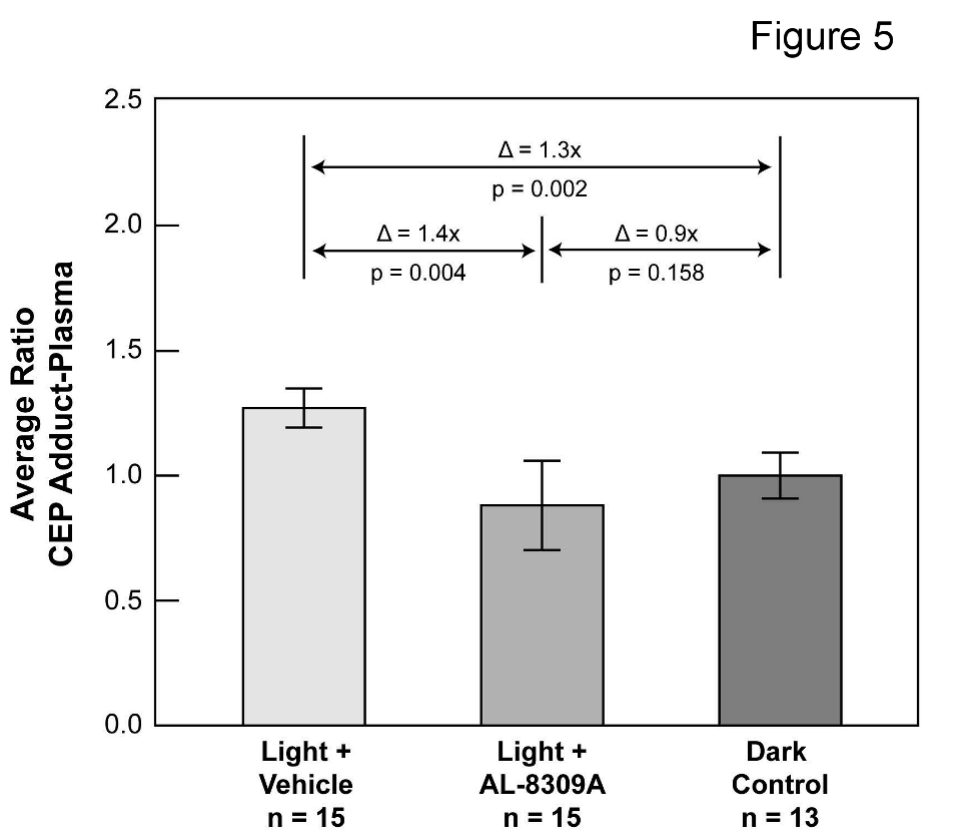
Pretreatment with AL-8309A reduces light-induced CEP immunoreactivity in rat plasma. A summary is shown of average CEP adduct levels in rat plasma following *in* *vivo* blue light exposure with or without pretreatment with AL-8309A (from Western analyses in Figure S4). Log_10_ transformed CEP optical density measurements were normalized to the mean dark control per analysis and average ratios transformed to linear scale. Δ represents the difference in average ratios between animals groups, the p-values are from the 2-sided t-test and error bars reflect standard deviation. The number of animals assayed in each group is indicated (n).

ELISA methods were not informative for the analysis of CEP in plasma from light exposed rats due to over saturation. Western blot analysis of rat plasma from light-exposed animals was useful for the analysis of CEP because high molecular weight components were not over saturated. The relative amounts of CEP adducts in plasma were estimated by densitometric analysis of high molecular weight CEP immunoreactive gel bands as illustrated in Figure 4 and Figure S3. This methodology has previously corroborated elevated CEP adducts in AMD plasma, and demonstrated reasonable agreement between the levels of high and low molecular weight CEP components in human plasma [22]. Western analyses of CEP adducts in plasma from 15 rats each with or without pretreatment with AL-8309A are itemized in Figure S4 and summarized in Figure 5. No outlier measurements were detected, all three datasets exhibited a normal distribution by the Shapiro-Wilk Normality Test and their means were significantly different by ANOVA (F = 6.2, F critical = 3.2, *p* = 0.005). Data variability was 9.2% RSD for the dark control group, 19.9% RSD for the light + drug group, and 6.3% RSD for the light + vehicle group. The results demonstrate that CEP immunoreactivity in plasma was elevated ~30% (t-test *p* < 0.002) in light exposed, vehicle-treated rats (n = 15) relative to dark controls (n = 13). Plasma CEP immunoreactivity was not elevated in light exposed, AL-8309A treated animals (n = 15). Compared to light + vehicle treated rats, pretreatment with AL-8309A reduced average CEP immunoreactivity in plasma ~30-40% (t-test *p* = 0.004). AL-8309A treatment of dark control animals reduced the average level of CEP adducts in rat plasma ~20% (*p* < 0.01) relative to the untreated dark control (Table S1).

### Pretreatment with AL-8309A reduces light-induced CEP autoantibody titers in rat plasma.

**Figure 6:**
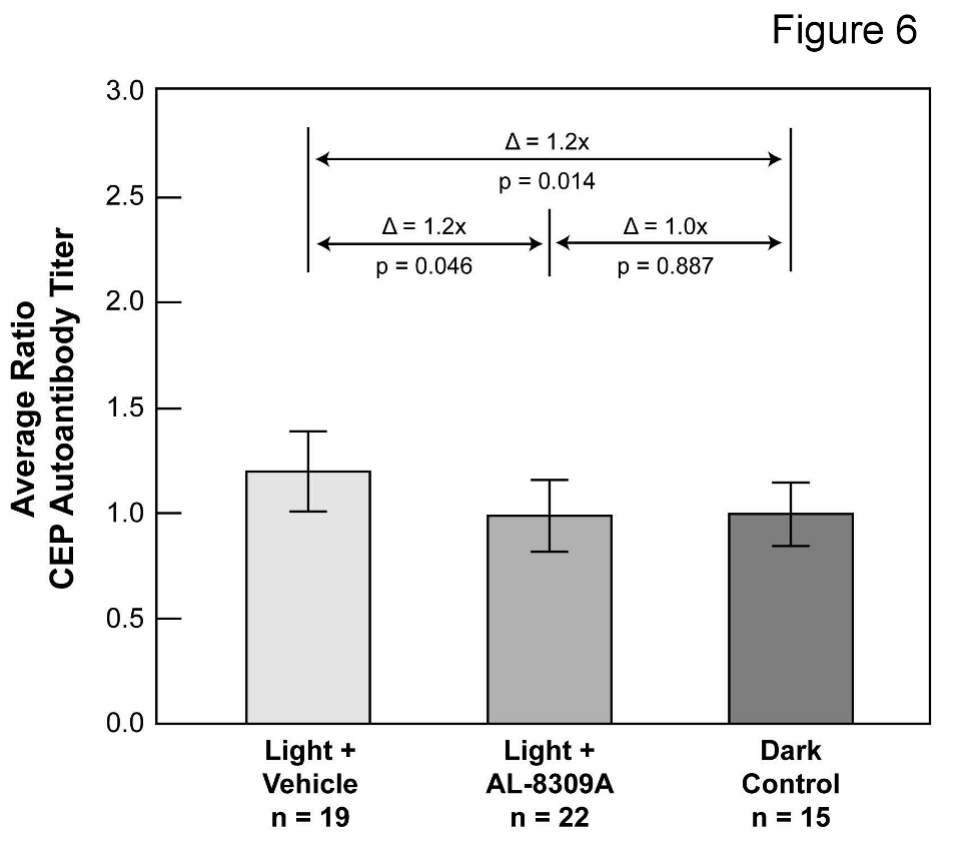
Pretreatment with AL-8309A reduces light-induced CEP autoantibody titers in rat plasma. A summary is shown of CEP autoantibody titer in rat plasma following in vivo blue light exposure with or without pretreatment with AL-8309A (from ELISA in Table S2). Log10 transformed CEP autoantibody titer measurements were normalized to the mean dark control per analysis and average ratios transformed to linear scale. Δ represents the difference in average ratios between animals groups, the p-values are from the 2-sided t-test and error bars reflect standard deviation. The number of animals assayed in each group is indicated (n).

ELISA was used to quantify CEP autoantibodies in plasma following light exposure with or without pretreatment with AL-8309A as detailed in Table S2. Figure 6 presents a summary of the normalized ELISA results after removal of outlier measurements (~5.4% of 111 total measurements). Outlier elimination reduced dark control animals from 16 to 15 and light + vehicle animals from 20 to 19 but did not impact the number of animals in the light + drug group (n = 22). All three datasets exhibited a normal distribution by the Shapiro-Wilk Normality Test and their means were significantly different by ANOVA (F = 3.30, F critical = 3.09, p < 0.04). Data variability was 15.4% RSD for the dark control group (including outliers), and 17.4% RSD for the light + drug group, and 15.8% RSD for the light + vehicle group (both excluding outliers). Relative to the dark control, the average CEP autoantibody titer was elevated ~20% (t-test *p* = 0.014) in light exposed rats without drug treatment and unchanged in rats pretreated with AL-8309A (p = 0.89). Compared with light + vehicle treated animals, light exposed rats pretreated with AL-8309A exhibited ~20% lower CEP autoantibody titer (t-test *p* = 0.046). This result does not satisfy the Bonferroni adjusted criteria of significance of p ≤ 0.017 and warrants further investigation. Treatment of dark control animals with AL-8309A had no apparent effect on CEP autoantibody titer in rat plasma (Table S1).

## Discussion

Previous *in vivo* studies have shown that intense light exposure increases CEP adducts in rat retina [24,25]. The purpose of this study was to determine whether light-induced CEP biomarkers are altered in rats treated with a drug shown to prevent light-induced retinal degeneration. Toward this goal, we quantified CEP adducts and autoantibodies in retina and plasma from rats exposed to intense blue light with and without pretreatment with AL-8309A, a serotonin 5-HT_1A_ receptor agonist that prevents light-induced structural and functional retinal damage [25]. Relative to dark control animals, light exposure resulted in the significant elevation of CEP adducts in retina and plasma and of CEP autoantibodies in plasma. Treatment with AL-8309A prior to light exposure significantly reduced the level of light-induced CEP adducts in rat retina and prevented elevation of CEP adducts and autoantibodies in plasma. Treatment with AL-8309A in the absence of light exposure reduced plasma CEP adduct levels but had no apparent effect on the basal levels of CEP adducts in retina or CEP autoantibodies in plasma. The current results are consistent with previous studies showing AL-8309A provides protection against photo-oxidative damage to the retina [25] and provide the first direct evidence that CEP biomarkers may be useful for monitoring the efficacy of therapeutics that enhance cellular defense to oxidative stress.

This study supports CEP biomarkers as potential tools for monitoring select therapeutics, however additional studies are warranted to better define the extent and consistency of CEP adduct and autoantibody changes in response to drug treatments. Statistical variability over all the data in this study ranged from 6.3%-21.6% RSD with about 4-5% of the measurements associated with Figure 2 and Figure 6 excluded as outliers. Sources of variability include technical variability in the Western blot and ELISA assays, as discussed previously [22], in animal handling/sample preparation and biological diversity. Despite the variability, ANOVA supported significant differences in mean amounts of CEP adducts and autoantibodies among the three experimental groups from retina and plasma. The t-test reinforced significant differences in CEP adduct levels in retina and plasma between specific pairs of groups based on a Bonferroni adjusted criteria of significance of *p* ≤ 0.017. The t-test also supported significant differences in autoantibody titer between the dark control and the light + vehicle groups but not between the light + vehicle and light + drug groups (*p* = 0.046). Additional analyses are required to clarify this issue. Furthermore, additional analyses are needed to address the controversial immunological question of whether extensive antibody production in the rat is possible within the 6h time frame used for light exposure. The present results support this possibility but more research is required to establish the immunological mechanisms.

CEP adducts are generated by covalent adduction of primary amino groups (e.g., protein ε-lysyl NH_2_) with 4-hydroxy-7-oxohept-5-enoic acid, an oxidative cleavage fragment derived *uniquely* from DHA-containing lipids [38]. DHA is the most oxidizable of all fatty acids and although present in very low amounts in most tissues and plasma, its highest abundancy in humans is in the retina [39], where it is concentrated in the photoreceptor rod outer segments and the RPE [40,41]. Notably, CEP adducts are elevated in AMD ocular tissues [5], stimulate neovascularization *in vivo* [18,19] and have been implicated in the development of dry AMD [20]. CEP adducts stimulate angiogenesis through toll-like receptor 2 (TLR2) and are hypothesized to contribute to wound healing/tissue repair in low concentrations, but at high concentrations may serve as catalysts, amplifying TLR2 signaling to promote excessive angiogenesis [19]. Mice immunized with CEP adducted albumin develop focal changes in the RPE resembling those in geographic atrophy, with animals with the most severe lesions also exhibiting significantly elevated CEP autoantibody titer [20]. CEP immunized mice also exhibit monocyte and macrophage migration into the interphotoreceptor matrix and elevated complement deposition in Bruch’s membrane [20]. CEP adducts and autoantibodies are elevated in AMD plasma [21,22], and are under study as biomarkers for assessing AMD risk in combination with genomic AMD markers [22]. Other oxidative modifications have been associated with AMD and may also offer AMD biomarker potential [4].

Serotonin regulates a variety of physiological functions and serotonin 5-HT_1A_ agonists have been used for the treatment of human anxiety disorders [42,43]. The mechanisms of action of 5-HT_1A_ receptor agonists are incompletely understood but such agonists appear to be neuroprotective in animal models of CNS ischemia [44,45], traumatic brain injury [46,47] and excitotoxicity [48,49]. The 5-HT_1A_ receptor reportedly activates the ERK pathway, leading to caspase 3 inhibition [50]. In other CNS systems, 5-HT_1A_ agonists have been reported to activate the mitogen-activated protein kinase (MAPK/ERK) signaling pathway, also leading to increased expression of anti-oxidant and anti-apoptotic proteins (i.e., SOD-1, catalase, Bcl-2, Bcl-XL, XIAP) [50–55].

While major differences exist in the pathogenesis of AMD and light-induced retinopathy, common features include oxidative damage as evidenced by elevated CEP adducts and autoantibodies in ocular tissues and plasma, and degeneration of the choroid, RPE and photoreceptors [56]. Recent studies demonstrated additional similarities, including light-induced complement deposition (i.e., C3, Factor B, Factor H and MAC) and microglia activation in the outer retina [26] of the blue light damage rat model. Treatment with AL-8309A not only prevents light-induced damage to retinal morphology and ERG responses [25] but also decreases microglia migration into and complement deposition in the outer retina [26]. Collectively, studies of AL-8309A using the retinal light damage model suggest this drug promotes cellular defense mechanisms that limit CEP adduct formation in retina, and prevent morphological damage and immune/inflammatory responses to oxidative stress in the retina. This study supports CEP biomarker efficacy in monitoring pharmacological interventions to acute retinal injury. Since CEP biomarkers are elevated in AMD plasma [22], they may also be useful for monitoring select therapies for chronic retinal injury.

## Supporting Information

Figure S1 **Coomassie Blue Staining as Loading Control for Figure 1.** Statistical analyses of sample amounts in Figure 1.
(PDF)

Figure S2 **CEP immunoreactivity in rat retina.** Western blot results following light exposure with or without AL-8309A treatment.
(PDF)

Figure S3 **Coomassie Blue Staining as Loading Control for Figure 4.** Statistical analyses of sample amounts in Figure 4.
(PDF)

Figure S4 **CEP immunoreactivity in rat plasma.** Western blot results following light exposure with or without AL-8309A treatment.
(PDF)

Table S1 **Impact of AL-8309A on Basal Levels of CEP Biomarkers.** Western blot results from dark controls with or without AL-8309A treatment.
(PDF)

Table S2 **CEP Autoantibody Titers.** ELISA results following light exposure with or without AL-8309A treatment.
(PDF)
