# Supplementary material for: CEP Biomarkers as Potential Tools for Monitoring Therapeutics
Source: PLoS One. 2013 Oct 1;8(10):e76325. doi: 10.1371/journal.pone.0076325 (PMC3788138; doi:10.1371/journal.pone.0076325)
Supplement: Figure S1 — Coomassie Blue Staining as Loading Control for Figure 1. Statistical analyses of sample amounts in Figure 1. (PDF) [file pone.0076325.s001.pdf]

A

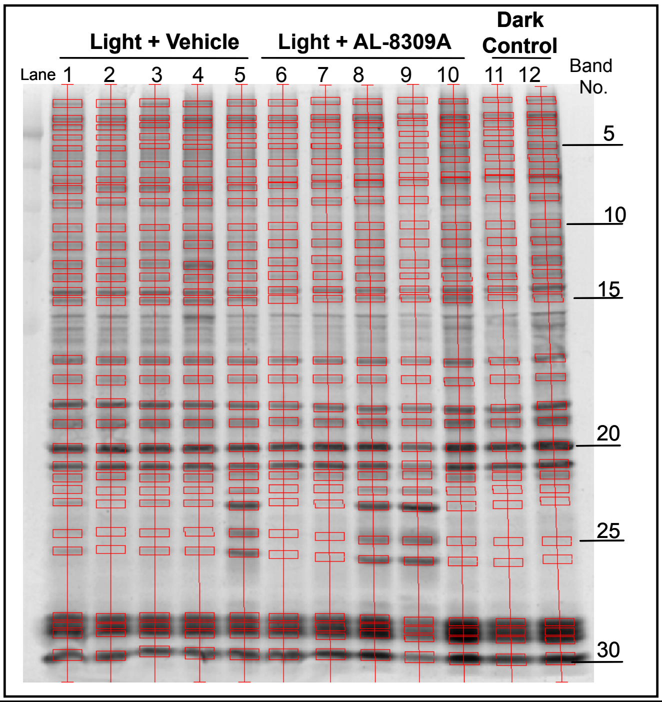

B

Quantity by Coomassie Blue Staining Intensity

Band	Light + Vehicle					Light + AL-8309A					Dark Control	
	1	2	3	4	5	6	7	8	9	10	11	12
1	48.20	44.63	47.03	45.42	44.85	41.84	44.27	45.28	48.31	45.15	48.73	46.41
2	56.45	55.00	51.56	55.33	49.83	43.43	45.84	54.65	51.50	58.53	49.73	55.21
3	43.83	42.63	49.68	53.14	46.56	41.08	51.14	48.60	34.84	53.54	45.74	59.55
4	38.99	37.97	41.91	45.08	38.01	35.55	36.35	42.09	40.07	47.31	39.79	44.60
5	40.32	40.05	41.61	45.55	37.38	36.75	37.60	42.66	41.73	40.91	44.51	45.60
6	46.29	45.00	49.61	51.12	52.44	40.56	41.20	48.21	44.78	45.67	40.16	44.16
7	51.71	50.43	46.49	47.07	49.06	46.96	46.16	50.08	41.69	44.59	41.43	45.22
8	55.96	55.78	59.28	52.28	52.05	50.23	58.82	58.08	54.06	55.02	51.64	58.05
9	55.26	52.48	55.36	59.97	50.71	48.39	50.09	54.81	40.75	53.47	53.25	54.82
10	51.93	49.97	45.17	53.64	46.12	43.88	46.05	49.77	41.60	49.09	50.13	51.16
11	69.40	67.62	69.28	62.28	61.77	58.79	61.06	66.95	64.63	63.03	63.78	65.80
12	63.56	66.09	69.47	68.78	62.56	56.22	57.26	65.17	67.69	67.43	65.87	62.70
13	61.40	64.36	64.36	63.43	64.94	56.96	56.36	63.32	63.31	67.89	63.97	67.41
14	67.28	68.80	69.67	63.78	64.72	58.53	55.84	58.89	66.66	66.49	66.98	71.16
15	63.28	65.45	61.84	68.46	63.63	57.44	64.04	57.63	56.16	58.02	61.12	60.83
16	71.71	76.89	78.75	77.64	70.38	63.33	61.47	76.70	63.45	77.97	73.86	73.68
17	60.29	64.07	65.95	64.00	58.59	62.03	54.05	58.69	60.74	65.73	62.54	61.43
18	86.56	88.76	86.11	89.08	83.54	85.19	82.46	86.38	90.54	90.15	81.95	82.97
19	83.98	82.76	88.38	85.08	76.87	69.06	79.60	76.07	82.11	86.08	89.21	89.41
20	121.16	122.81	123.56	112.15	118.41	113.94	117.35	122.29	112.69	117.18	116.23	115.65
21	93.91	97.97	96.41	97.03	93.76	88.33	100.54	89.52	88.54	91.91	98.91	102.54
22	64.17	57.94	63.96	64.33	67.21	67.07	62.13	65.63	63.19	67.96	65.48	68.25
23	43.95	46.16	47.34	42.25	46.74	40.05	44.37	48.11	48.34	45.68	48.77	48.18
24	50.69	41.74	46.50	43.21	102.74	45.54	42.46	103.38	124.12	52.76	41.86	40.89
25	51.48	42.29	48.64	45.78	71.23	42.75	44.09	80.80	87.49	56.14	42.84	45.81
26	57.56	54.28	50.77	49.35	77.02	49.38	47.17	77.99	96.89	54.03	46.28	43.90
27	96.21	109.47	89.85	89.96	86.73	86.90	90.18	103.07	89.83	132.69	116.97	113.57
28	92.34	80.31	94.68	93.94	107.44	90.31	97.43	91.59	80.20	143.73	118.11	133.84
29	97.79	85.41	95.97	98.90	94.84	97.34	94.91	130.34	83.71	131.74	107.85	118.28
30	137.32	135.10	133.20	137.29	144.83	141.94	148.76	156.62	111.73	171.07	153.70	179.69
**Lane Totals**	2022.98	1992.23	2032.37	2025.32	2084.94	1859.75	1919.05	2173.36	2041.34	2200.96	2051.37	2150.75
**Group Average**			2031.57					2038.69			2101.06	
**SD**			33.58					150.67			70.27	
**RSD**			1.65%					7.39%			3.34%	

0.11 = *p*-value [Light + Vehicle] vs [Dark Control]
0.61 = *p*-value [Light + AL-8309A] vs [Dark Control]
0.92 = *p*-value [Light+Vehicle] vs [Light+AL-8309A]

Supporting Figure S1. Coomassie Blue Staining as Loading Control for Figure 1.
(A) Coomassie blue staining intensity of the indicated SDS-PAGE bands in hardcopy Figure 1B was quantified using Quantity One software (BioRad). **(B)** The quantity of each highlighted band is listed along with the sum total per lane, as well as the average total lane quantity, standard deviation (SD) and relative standard deviation (RSD) for the groups Light + Vehicle, Light + AL-8309A, Dark Control. Also shown are p-values (2 sided t-test) for comparison of total lane quantities between the three experimental groups. The statistical analyses reveal relatively low variability and no significant differences in total staining intensity between the experimental groups. Overall, Coomassie blue staining intensity supports approximately equal amounts of protein applied per lane.
