# Supplementary material for: CEP Biomarkers as Potential Tools for Monitoring Therapeutics
Source: PLoS One. 2013 Oct 1;8(10):e76325. doi: 10.1371/journal.pone.0076325 (PMC3788138; doi:10.1371/journal.pone.0076325)
Supplement: Figure S2 — CEP immunoreactivity in rat retina. Western blot results following light exposure with or without AL-8309A treatment. (PDF) [file pone.0076325.s002.pdf]

Supporting Figure S2

CEP immunoreactivity in rat retina. Western blots of retinal homogenates with anti-CEP monoclonal antibody are shown from blue light exposed rats pretreated with AL-8309A or vehicle, and from untreated dark control animals. Each lane represents one animal. Results from three separate animal experiments are shown: Experiment 1 (A-G), Experiment 2 (H-O), and Experiment 3 (P,Q). CEP immunoreactivity was quantified by densitometry as shown in the graphs where error bars represent standard deviation. The number of animals assayed in each group is indicated (n). CEP modified human serum albumin (CEP-HSA) was used as a positive control. Overall average results are presented in hardcopy Figure 2.

Fig S2 AB

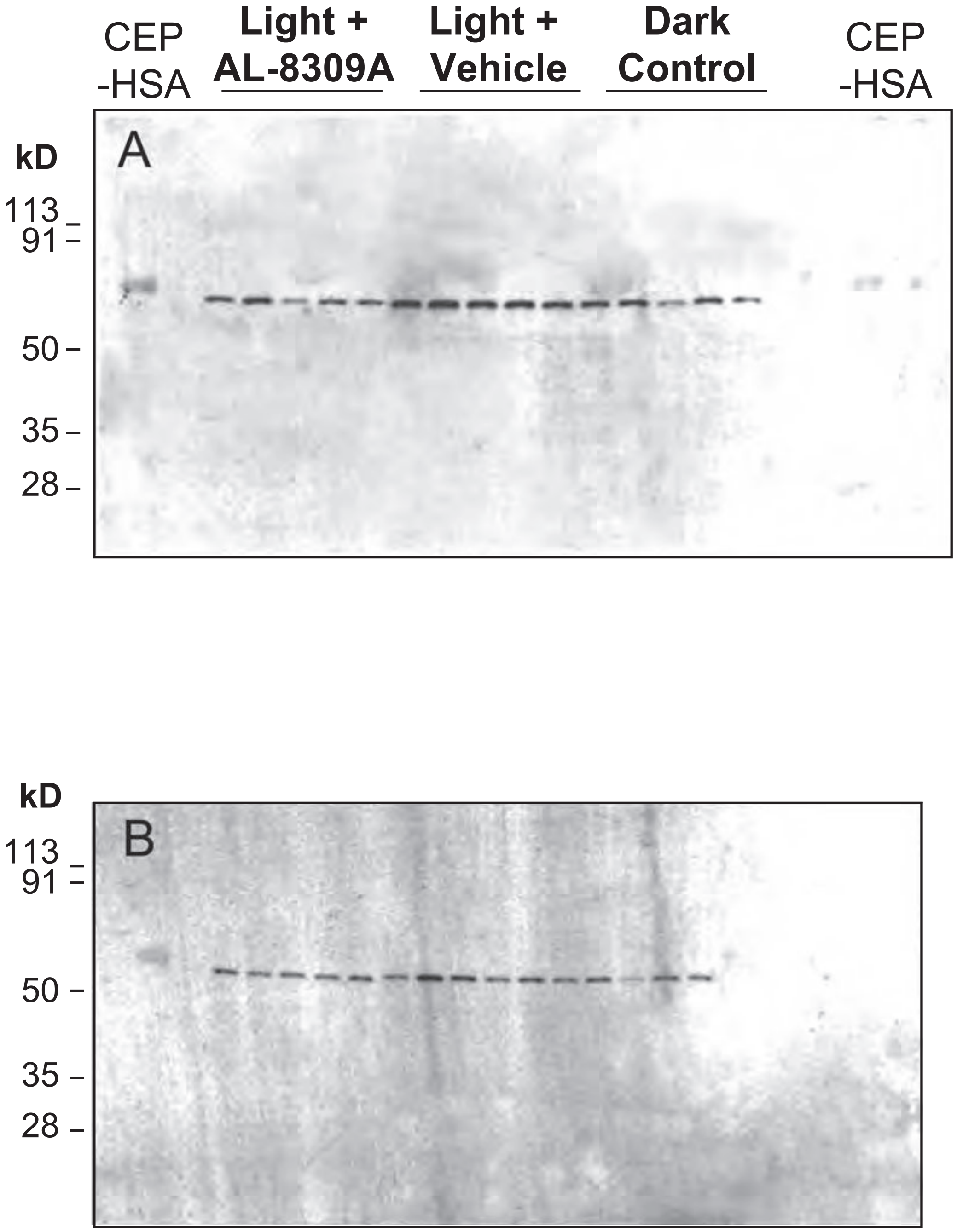
CEP -HSA
Light + AL-8309A
Light + Vehicle
Dark Control
CEP -HSA
kD
A
113
91
50
35
28
kD
B
113
91
50
35
28

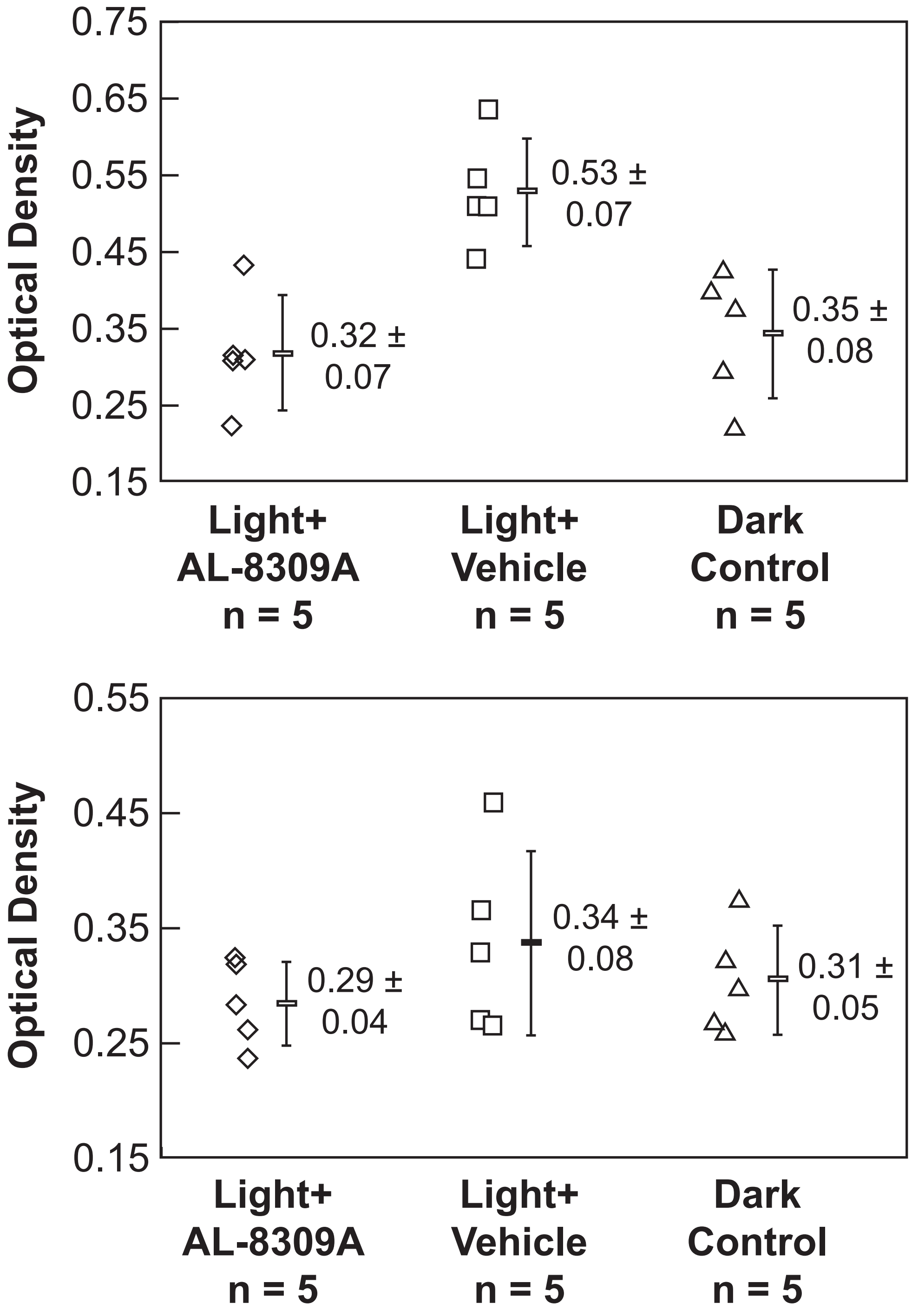
Optical Density
0.75
0.65
0.55
0.45
0.35
0.25
0.15
0.32 ± 0.07
0.53 ± 0.07
0.35 ± 0.08
Light+ AL-8309A n = 5
Light+ Vehicle n = 5
Dark Control n = 5
Optical Density
0.55
0.45
0.35
0.25
0.15
0.29 ± 0.04
0.34 ± 0.08
0.31 ± 0.05
Light+ AL-8309A n = 5
Light+ Vehicle n = 5
Dark Control n = 5

Fig S2 CD

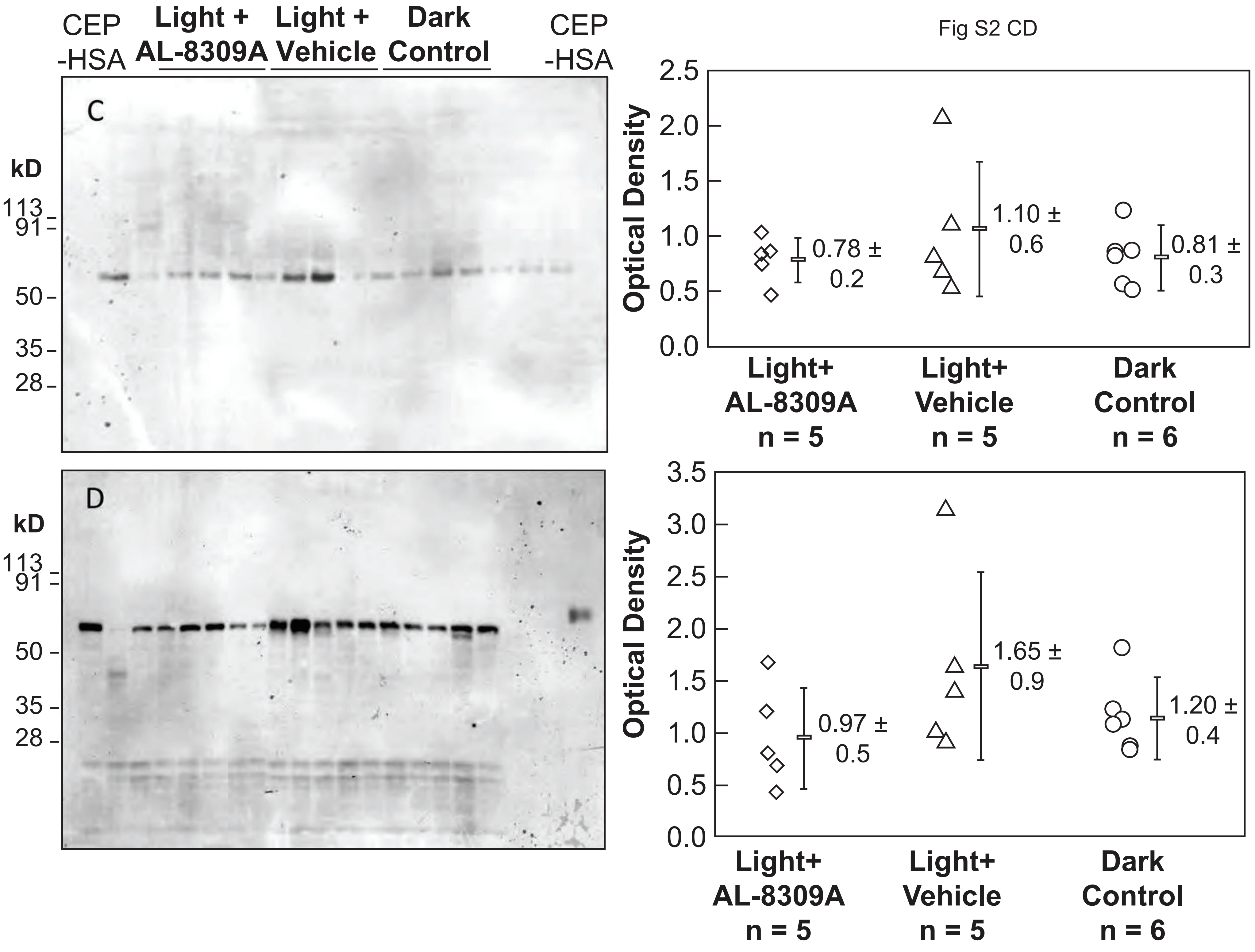

CEP
-HSA
Light +
AL-8309A
Light +
Vehicle
Dark
Control
CEP
-HSA
C
kD
113
91
50
35
28
D
kD
113
91
50
35
28
Optical Density
2.5
2.0
1.5
1.0
0.5
0.0
0.78 ±
0.2
1.10 ±
0.6
0.81 ±
0.3
Light+
AL-8309A
n = 5
Light+
Vehicle
n = 5
Dark
Control
n = 6
Optical Density
3.5
3.0
2.5
2.0
1.5
1.0
0.5
0.0
0.97 ±
0.5
1.65 ±
0.9
1.20 ±
0.4
Light+
AL-8309A
n = 5
Light+
Vehicle
n = 5
Dark
Control
n = 6

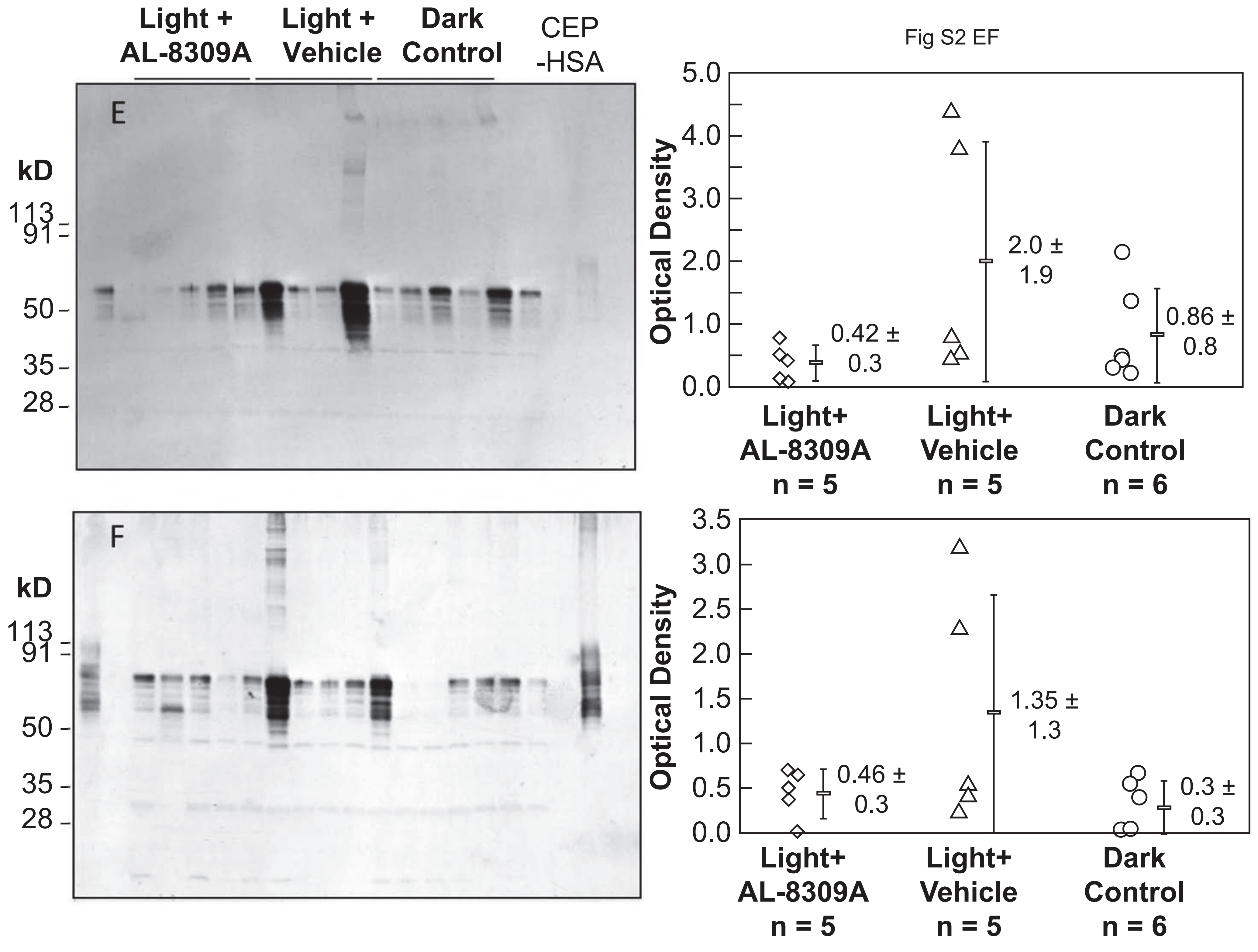
Fig S2 EF
Light + AL-8309A
Light + Vehicle
Dark Control
CEP -HSA
E
kD
113
91
50
35
28
Optical Density
5.0
4.0
3.0
2.0
1.0
0.0
0.42 ± 0.3
2.0 ± 1.9
0.86 ± 0.8
Light+ AL-8309A n = 5
Light+ Vehicle n = 5
Dark Control n = 6
F
kD
113
91
50
35
28
Optical Density
3.5
3.0
2.5
2.0
1.5
1.0
0.5
0.0
0.46 ± 0.3
1.35 ± 1.3
0.3 ± 0.3
Light+ AL-8309A n = 5
Light+ Vehicle n = 5
Dark Control n = 6

Fig S2 G

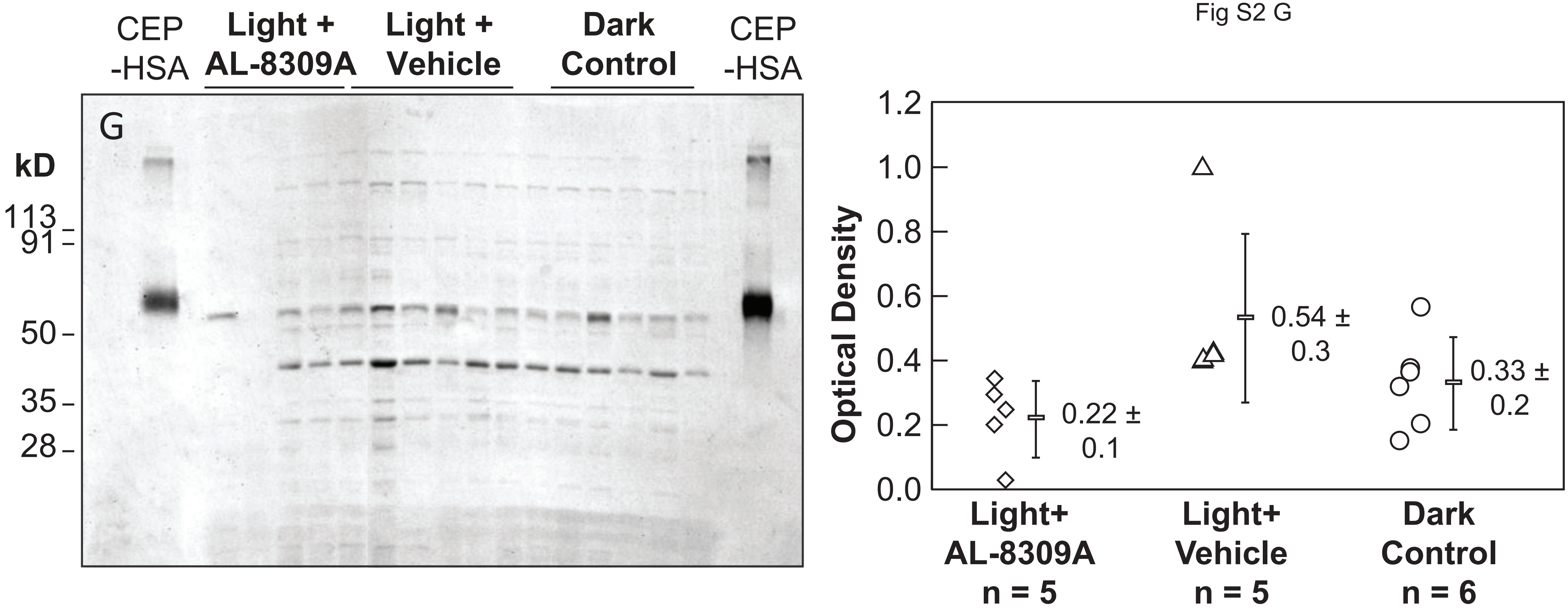
CEP
-HSA
Light +
AL-8309A
Light +
Vehicle
Dark
Control
CEP
-HSA
G
kD
113
91
50
35
28
1.2
1.0
0.8
0.6
0.4
0.2
0.0
Optical Density
0.22 ±
0.1
0.54 ±
0.3
0.33 ±
0.2
Light+
AL-8309A
n = 5
Light+
Vehicle
n = 5
Dark
Control
n = 6

Fig S2 HI

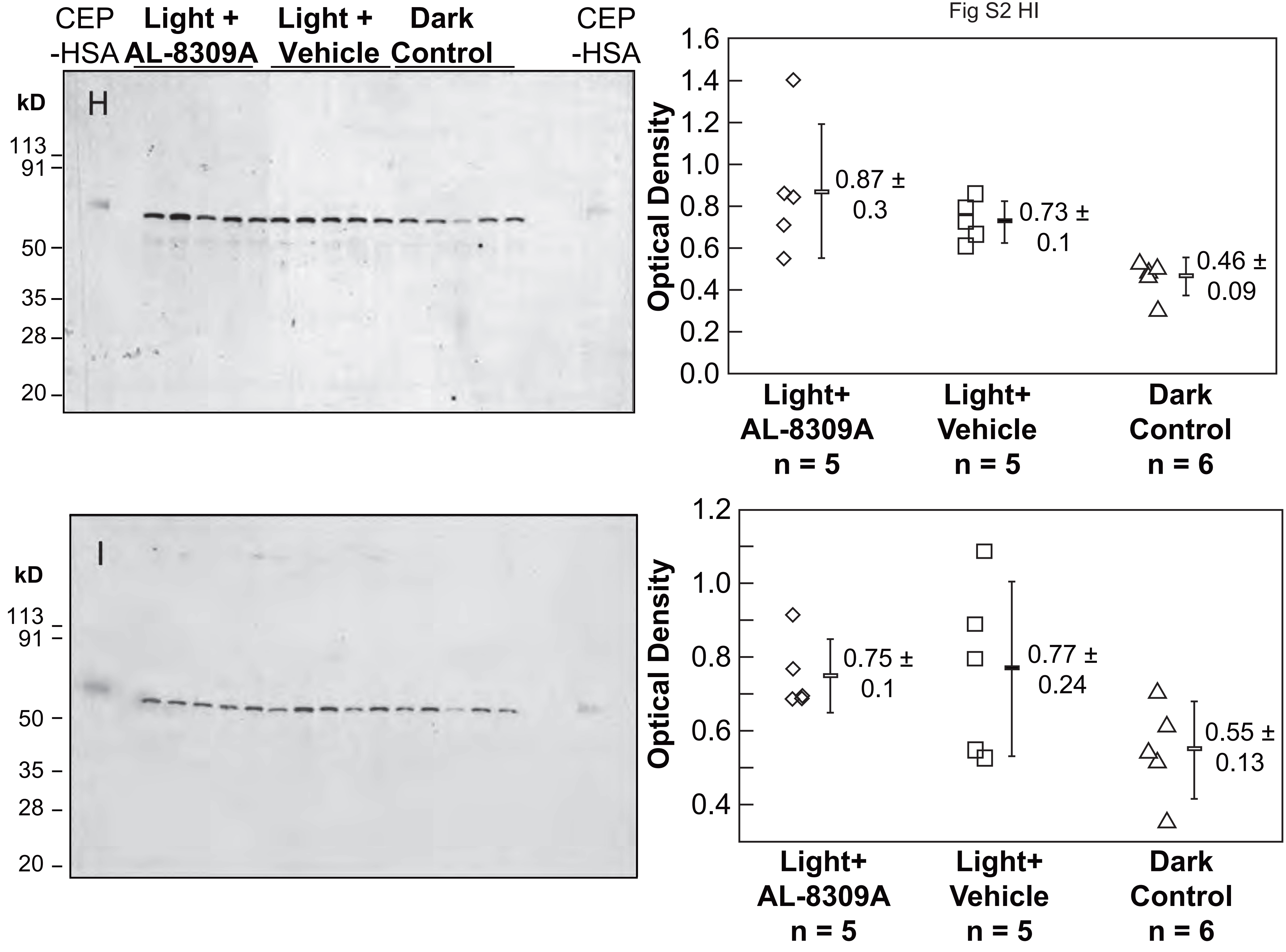

CEP -HSA
Light + AL-8309A
Light + Vehicle
Dark Control
CEP -HSA
H
kD
113
91
50
35
28
20
1.6
1.4
1.2
1.0
0.8
0.6
0.4
0.2
0.0
Optical Density
0.87 ± 0.3
0.73 ± 0.1
0.46 ± 0.09
Light+ AL-8309A n = 5
Light+ Vehicle n = 5
Dark Control n = 6
I
kD
113
91
50
35
28
20
1.2
1.0
0.8
0.6
0.4
Optical Density
0.75 ± 0.1
0.77 ± 0.24
0.55 ± 0.13
Light+ AL-8309A n = 5
Light+ Vehicle n = 5
Dark Control n = 6

Fig S2 JK

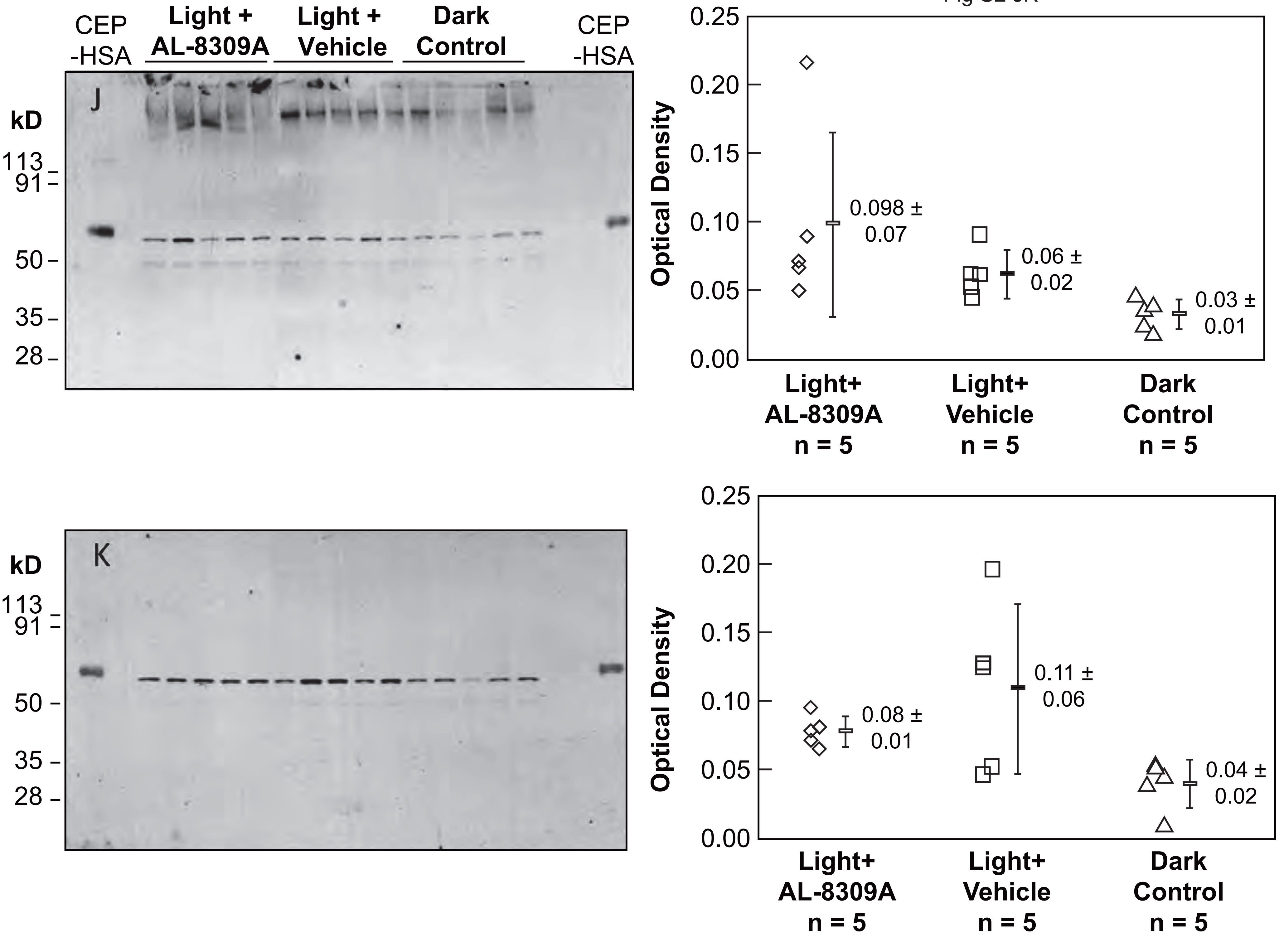

CEP
-HSA
Light +
AL-8309A
Light +
Vehicle
Dark
Control
CEP
-HSA
J
kD
113
91
50
35
28
Optical Density
0.25
0.20
0.15
0.10
0.05
0.00
0.098 ±
0.07
0.06 ±
0.02
0.03 ±
0.01
Light+
AL-8309A
n = 5
Light+
Vehicle
n = 5
Dark
Control
n = 5
K
kD
113
91
50
35
28
Optical Density
0.25
0.20
0.15
0.10
0.05
0.00
0.08 ±
0.01
0.11 ±
0.06
0.04 ±
0.02
Light+
AL-8309A
n = 5
Light+
Vehicle
n = 5
Dark
Control
n = 5

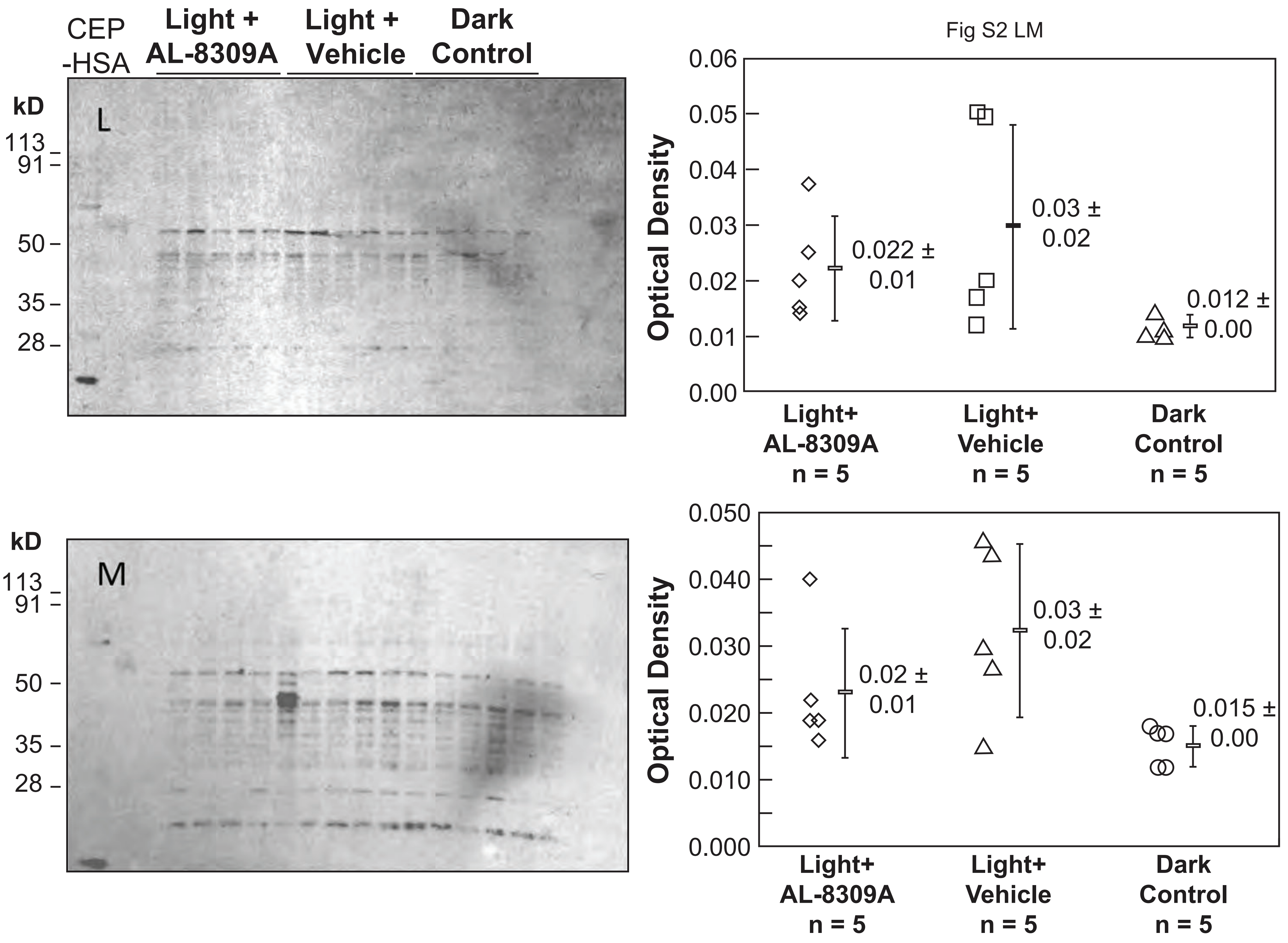

Fig S2 LM
CEP -HSA
Light + AL-8309A
Light + Vehicle
Dark Control
kD
L
113
91
50
35
28
Optical Density
0.06
0.05
0.04
0.03
0.02
0.01
0.00
0.022 ± 0.01
0.03 ± 0.02
0.012 ± 0.00
Light+ AL-8309A n = 5
Light+ Vehicle n = 5
Dark Control n = 5
kD
M
113
91
50
35
28
Optical Density
0.050
0.040
0.030
0.020
0.010
0.000
0.02 ± 0.01
0.03 ± 0.02
0.015 ± 0.00
Light+ AL-8309A n = 5
Light+ Vehicle n = 5
Dark Control n = 5

Fig S2 NO

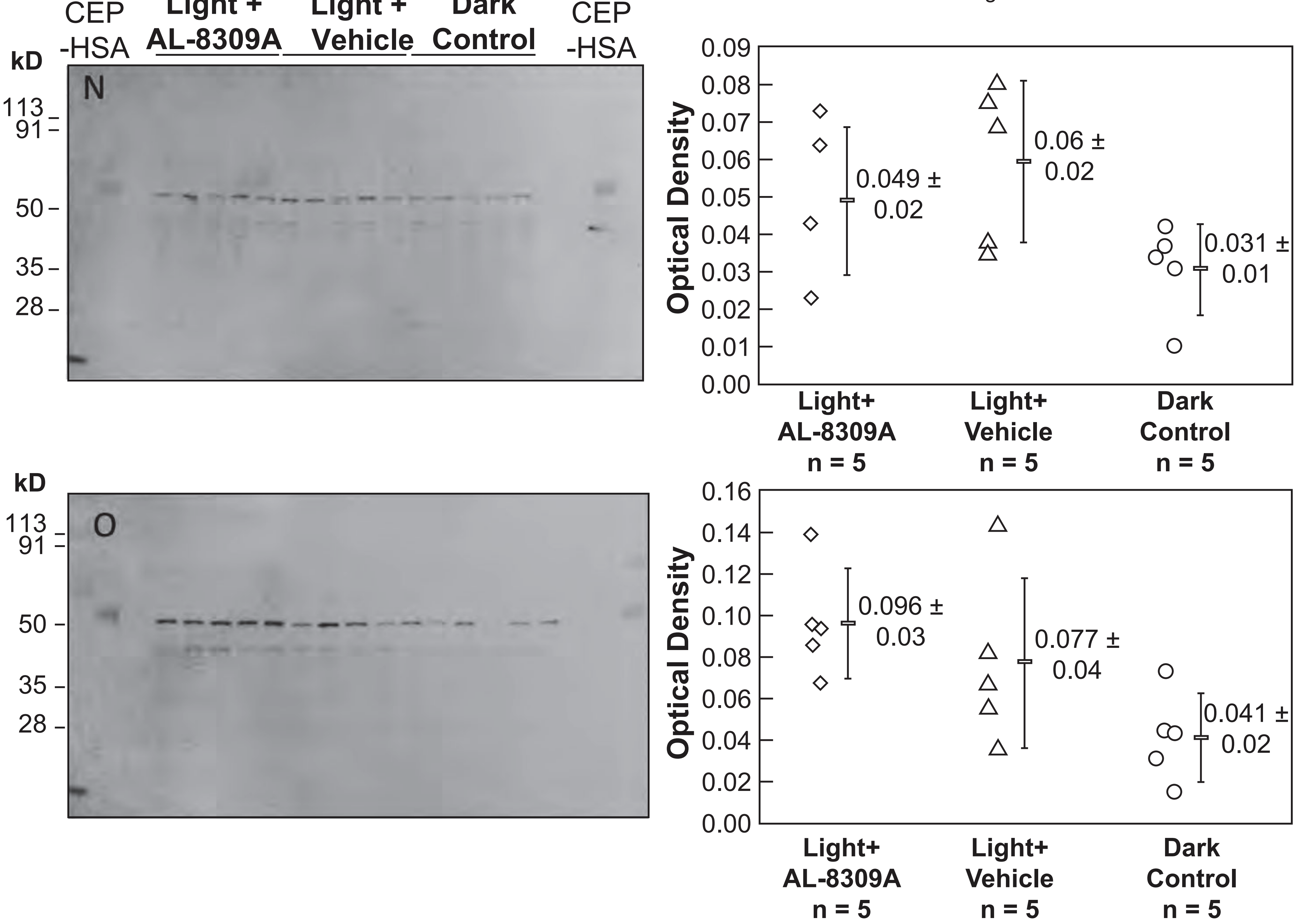

CEP
-HSA
Light +
AL-8309A
Light +
Vehicle
Dark
Control
CEP
-HSA
kD
N
113
91
50
35
28
O
113
91
50
35
28
Optical Density
0.09
0.08
0.07
0.06
0.05
0.04
0.03
0.02
0.01
0.00
0.049 ±
0.02
0.06 ±
0.02
0.031 ±
0.01
Light+
AL-8309A
n = 5
Light+
Vehicle
n = 5
Dark
Control
n = 5
0.16
0.14
0.12
0.10
0.08
0.06
0.04
0.02
0.00
0.096 ±
0.03
0.077 ±
0.04
0.041 ±
0.02

Fig S2 P

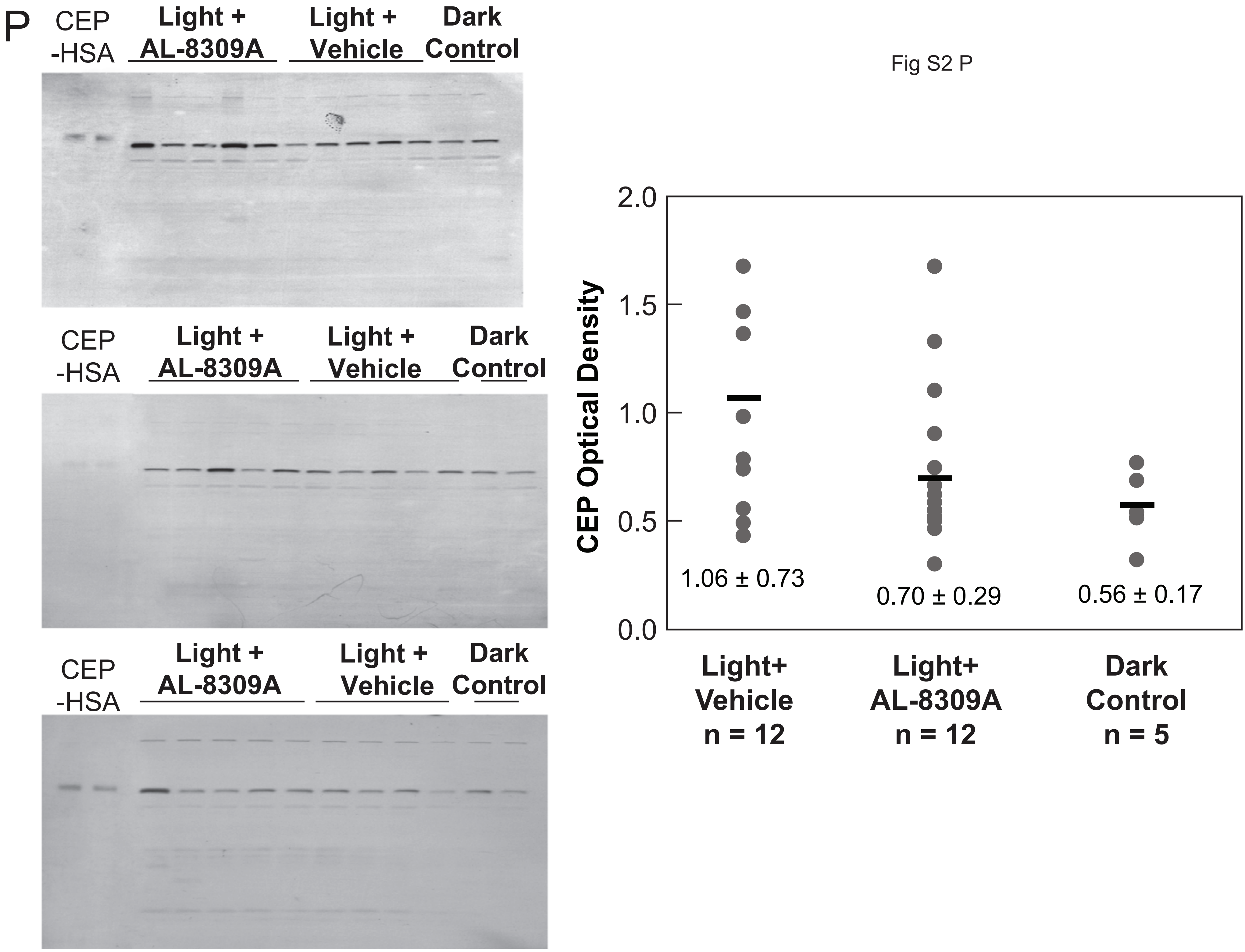
P
CEP -HSA
Light + AL-8309A
Light + Vehicle
Dark Control
CEP -HSA
Light + AL-8309A
Light + Vehicle
Dark Control
CEP -HSA
Light + AL-8309A
Light + Vehicle
Dark Control
CEP Optical Density
2.0
1.5
1.0
0.5
0.0
1.06 ± 0.73
0.70 ± 0.29
0.56 ± 0.17
Light+ Vehicle n = 12
Light+ AL-8309A n = 12
Dark Control n = 5

Fig S2 Q

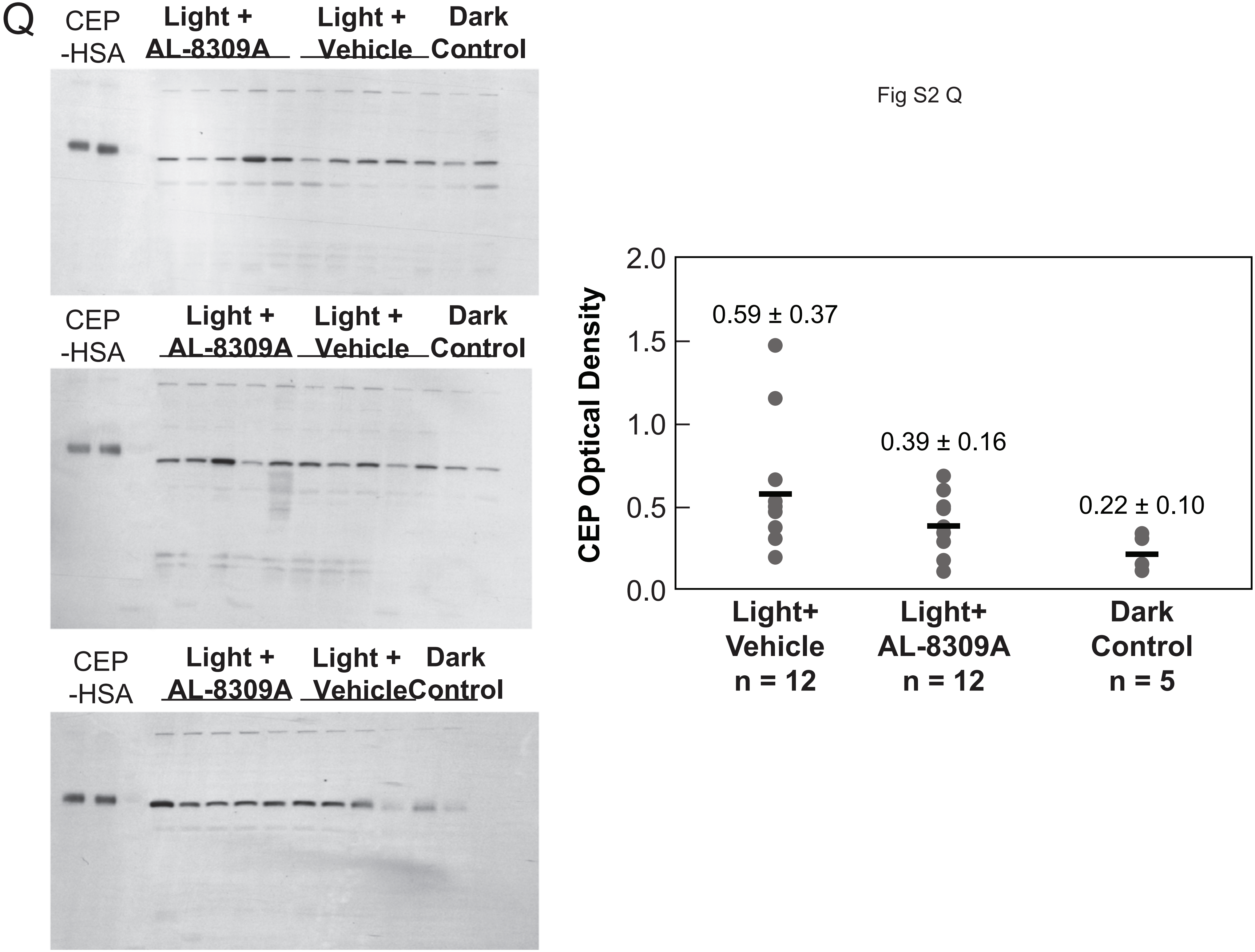
Q
CEP -HSA
Light + AL-8309A
Light + Vehicle
Dark Control
CEP Optical Density
2.0
1.5
1.0
0.5
0.0
0.59 ± 0.37
0.39 ± 0.16
0.22 ± 0.10
Light+ Vehicle n = 12
Light+ AL-8309A n = 12
Dark Control n = 5
