# Supplementary material for: CEP Biomarkers as Potential Tools for Monitoring Therapeutics
Source: PLoS One. 2013 Oct 1;8(10):e76325. doi: 10.1371/journal.pone.0076325 (PMC3788138; doi:10.1371/journal.pone.0076325)
Supplement: Figure S4 — CEP immunoreactivity in rat plasma. Western blot results following light exposure with or without AL-8309A treatment. (PDF) [file pone.0076325.s004.pdf]

Supporting Figure S4

CEP immunoreactivity in rat plasma. Western blots of rat plasma with anti-CEP monoclonal antibody are shown from blue light exposed rats pretreated with AL-8309A or vehicle, and from untreated dark control animals. Results from two separate animal experiments are shown: Experiment 1 (A,B), Experiment 2 (C). Each lane represents one animal. CEP immunoreactivity was quantified by densitometry as shown in the graphs where error bars represent standard deviation. CEP modified human serum albumin (CEP-HSA) was used as a positive control. The number of animals assayed in each group is indicated (n). Overall average results are presented in hardcopy Figure 5.

Fig S4 AB

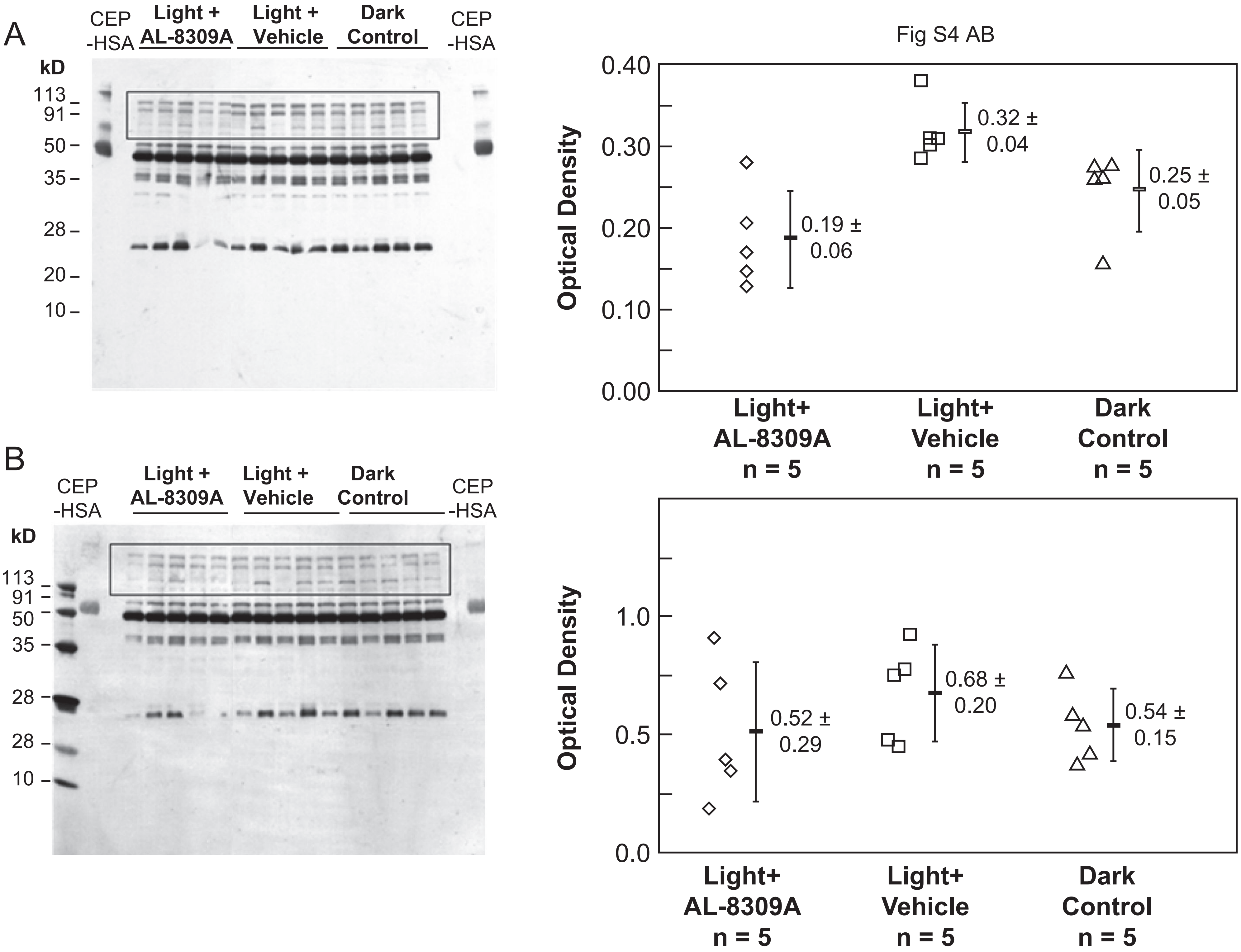

A
CEP -HSA
Light + AL-8309A
Light + Vehicle
Dark Control
CEP -HSA
kD
113
91
50
35
28
20
10
Optical Density
0.40
0.30
0.20
0.10
0.00
0.19 ± 0.06
0.32 ± 0.04
0.25 ± 0.05
Light+ AL-8309A n = 5
Light+ Vehicle n = 5
Dark Control n = 5
B
CEP -HSA
Light + AL-8309A
Light + Vehicle
Dark Control
CEP -HSA
kD
113
91
50
35
28
28
10
Optical Density
1.0
0.5
0.0
0.52 ± 0.29
0.68 ± 0.20
0.54 ± 0.15
Light+ AL-8309A n = 5
Light+ Vehicle n = 5
Dark Control n = 5

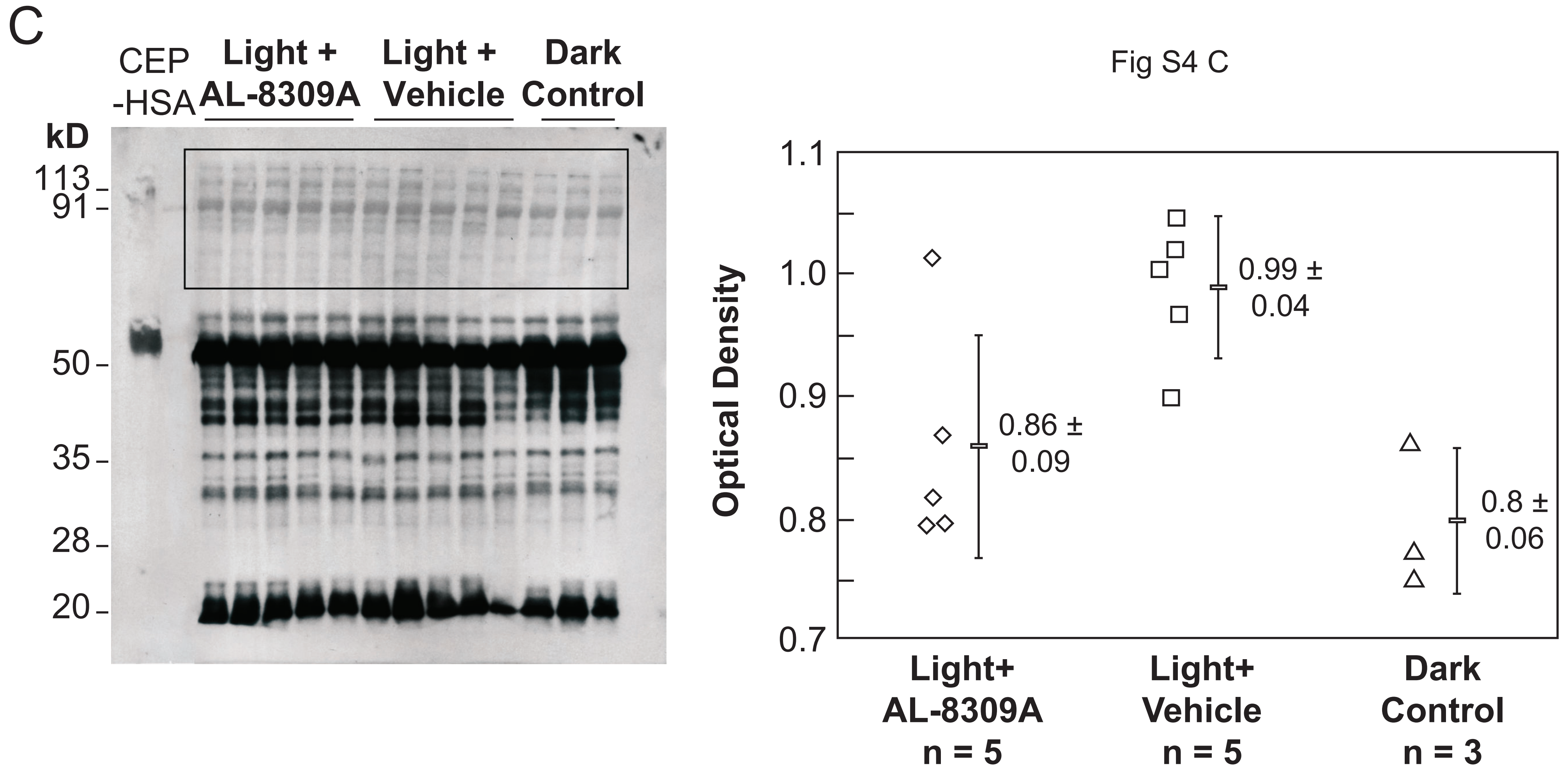
C
CEP
-HSA
Light +
AL-8309A
Light +
Vehicle
Dark
Control
kD
113
91
50
35
28
20
Fig S4 C
Optical Density
1.1
1.0
0.9
0.8
0.7
0.86 ±
0.09
0.99 ±
0.04
0.8 ±
0.06
Light+
AL-8309A
n = 5
Light+
Vehicle
n = 5
Dark
Control
n = 3
