# Supplementary material for: CEP Biomarkers as Potential Tools for Monitoring Therapeutics
Source: PLoS One. 2013 Oct 1;8(10):e76325. doi: 10.1371/journal.pone.0076325 (PMC3788138; doi:10.1371/journal.pone.0076325)
Supplement: Table S1 — Impact of AL-8309A on Basal Levels of CEP Biomarkers. Western blot results from dark controls with or without AL-8309A treatment. (PDF) [file pone.0076325.s005.pdf]

Supporting Table S1

Impact of AL-8309A on Basal Levels of CEP Biomarkers

	Dark Control + AL-8309A	Dark Control	***p* value** t-test
**CEP Adducts[1] Retina**	1.44 ± 0.15<br>n = 12	1.00 ± 0.20<br>n = 5	0.10
**CEP Adducts[1] Plasma**	0.80 ± 0.05<br>n = 10	1.00 ± 0.06<br>n = 6	<0.01
**CEP[2] Autoantibodies**	0.89 ± 0..22<br>n = 11	1.00 ± 0.11<br>n = 5	0.65

1. CEP adducts were quantified by Western analysis of retina or plasma from dark control animals treated or untreated with AL-8309A. Values reflect mean average optical density ± standard deviation. Mean values were calculated from log transformed data normalized to the mean dark control then transformed to linear scale.

2. Plasma CEP autoantibodies were measured by ELISA in dark control animals treated or untreated with AL-8309A. Values reflect mean titer ± standard deviation. Mean values were calculated from log transformed data normalized to the mean dark control then extrapolated to linear scale. The number of animals assayed is indicated (n). The p values reflect the significance of concentration differences between AL-8309A treated and untreated animals in the dark.
