# Supplementary material for: CEP Biomarkers as Potential Tools for Monitoring Therapeutics
Source: PLoS One. 2013 Oct 1;8(10):e76325. doi: 10.1371/journal.pone.0076325 (PMC3788138; doi:10.1371/journal.pone.0076325)
Supplement: Table S2 — CEP Autoantibody Titers. ELISA results following light exposure with or without AL-8309A treatment. (PDF) [file pone.0076325.s006.pdf]

Supporting Table S2
Log CEP Autoantibody Titer

Experiment	Light + AL8309A	Light + Vehicle	Dark control
Experiment 1 analysis 1	-0.317	0.147	0.039
	0.104	-0.212	0.039
	-0.058	-0.103	-0.103
	-0.058	-0.080	-0.182
	-0.080	-0.154	0.185
			0.022
Experiment 1 analysis 2	-0.269	0.233	0.152
	0.104	-0.135	-0.010
	-0.080	-0.032	-0.056
	-0.135	-0.056	-0.197
	-0.135	-0.080	0.121
			-0.010
Experiment 2 analysis 1	-0.166	0.533	[0.405]
	-0.099	[0.749]	0.026
	[0.513]	0.135	-0.079
	0.311	0.503	0.070
	-0.120	0.056	[-0.421]
Experiment 2 analysis 2	0.106	0.459	[0.285]
	0.285	[0.777]	-0.009
	0.421	0.153	-0.081
	0.095	0.250	-0.009
	-0.185	-0.097	-0.185
Experiment 3 analysis 1	-0.115	-0.122	0.023
	-0.088	0.310	0.057
	-0.175	0.034	0.112
	-0.058	0.182	-0.014
	0.008	0.003	-0.179
	-0.269	-0.034	
	-0.148	0.081	
	0.142	0.257	
	0.102	-0.070	
Experiment 3 analysis 2	0.132	-0.003	-0.057
	0.280	0.162	0.040
	-0.270	-0.007	0.104
	-0.006	-0.005	-0.086
	0.092	0.010	
	0.154	0.119	
	0.075	0.218	
	0.133	0.342	
	0.201	-0.180	
	-0.076	0.125	
	-0.018		
	0.005		
Mean (Log)	0.008	0.115	0.000
SD	0.189	0.240	0.154
Mean (Linear)	1.02	1.30	1.00
RSD	18.51%	18.42%	15.37%
n	22	20	16
**Outliers Removed**			
n	22	19	15
Mean (Log)	-0.004	0.079	-0.010
SD	0.173	0.190	0.102
Mean (Linear)	0.99	1.20	0.98
RSD	17.44%	15.81%	10.44%

CEP autoantibody titer in rat plasma was measured in three separate experiments by direct ELISA following *in vivo* blue light exposure with or without pretreatment with AL-8309A (ELISA method C in J Biol Chem 278, 42027). The titer was defined as the ratio of plasma binding to antigen (A) versus binding to BSA (A_o) where the antigen was the plate coating agent CEP-BSA. Log transformed data are shown after normalization to the dark control mean value per analysis. The number of animals (n), the standard deviation (SD) and the relative standard deviation (RSD) are indicated. Outliers were identified [values in brackets] using the interquartile method. Log mean values were transformed to linear scale.
